# Chromosome-level genome assembly and resequencing of camphor tree (*Cinnamomum camphora*) provides insight into phylogeny and diversification of terpenoid and triglyceride biosynthesis of *Cinnamomum*

**DOI:** 10.1093/hr/uhac216

**Published:** 2022-09-21

**Authors:** Xin-Dong Wang, Chun-Yan Xu, Yong-Jie Zheng, Yan-Fang Wu, Yue-Ting Zhang, Ting Zhang, Zhen-Yu Xiong, Hai-Kuan Yang, Jiang Li, Chao Fu, Feng-Ying Qiu, Xiao-Ying Dai, Xin-Liang Liu, Xiao-San He, Song-Song Zhou, Sheng-Xing Li, Tao Fu, Han Xie, Yan-Ling Chen, Qian-Qian Zhang, Hong-Qi Wang, Yang-Dong Wang, Cheng Zhou, Xiang-Mei Jiang

**Affiliations:** Camphor Engineering and Technology Research Center of National Forestry and Grassland Administration, Jiangxi Academy of Forestry, Nanchang 330032, China; Jiangxi Provincial Key Lab for Plant Biotechnology, Jiangxi Academy of Forestry, Nanchang 330032, Jiangxi, China; BGI Genomics, BGI-Shenzhen, Shenzhen 518083, China; Camphor Engineering and Technology Research Center of National Forestry and Grassland Administration, Jiangxi Academy of Forestry, Nanchang 330032, China; Research Institute of Subtropical Forestry, Chinese Academy of Forestry, Hangzhou 311400, China; Camphor Engineering and Technology Research Center of National Forestry and Grassland Administration, Jiangxi Academy of Forestry, Nanchang 330032, China; Camphor Engineering and Technology Research Center of National Forestry and Grassland Administration, Jiangxi Academy of Forestry, Nanchang 330032, China; Jiangxi Provincial Key Lab for Plant Biotechnology, Jiangxi Academy of Forestry, Nanchang 330032, Jiangxi, China; Camphor Engineering and Technology Research Center of National Forestry and Grassland Administration, Jiangxi Academy of Forestry, Nanchang 330032, China; Jiangxi Provincial Key Lab for Plant Biotechnology, Jiangxi Academy of Forestry, Nanchang 330032, Jiangxi, China; Camphor Engineering and Technology Research Center of National Forestry and Grassland Administration, Jiangxi Academy of Forestry, Nanchang 330032, China; Camphor Engineering and Technology Research Center of National Forestry and Grassland Administration, Jiangxi Academy of Forestry, Nanchang 330032, China; Camphor Engineering and Technology Research Center of National Forestry and Grassland Administration, Jiangxi Academy of Forestry, Nanchang 330032, China; Camphor Engineering and Technology Research Center of National Forestry and Grassland Administration, Jiangxi Academy of Forestry, Nanchang 330032, China; Camphor Engineering and Technology Research Center of National Forestry and Grassland Administration, Jiangxi Academy of Forestry, Nanchang 330032, China; Camphor Engineering and Technology Research Center of National Forestry and Grassland Administration, Jiangxi Academy of Forestry, Nanchang 330032, China; Camphor Engineering and Technology Research Center of National Forestry and Grassland Administration, Jiangxi Academy of Forestry, Nanchang 330032, China; Camphor Engineering and Technology Research Center of National Forestry and Grassland Administration, Jiangxi Academy of Forestry, Nanchang 330032, China; Camphor Engineering and Technology Research Center of National Forestry and Grassland Administration, Jiangxi Academy of Forestry, Nanchang 330032, China; BGI Genomics, BGI-Shenzhen, Shenzhen 518083, China; BGI Genomics, BGI-Shenzhen, Shenzhen 518083, China; BGI Genomics, BGI-Shenzhen, Shenzhen 518083, China; BGI Genomics, BGI-Shenzhen, Shenzhen 518083, China; BGI Genomics, BGI-Shenzhen, Shenzhen 518083, China; Research Institute of Subtropical Forestry, Chinese Academy of Forestry, Hangzhou 311400, China; Camphor Engineering and Technology Research Center of National Forestry and Grassland Administration, Jiangxi Academy of Forestry, Nanchang 330032, China; Camphor Engineering and Technology Research Center of National Forestry and Grassland Administration, Jiangxi Academy of Forestry, Nanchang 330032, China

## Abstract

*Cinnamomum* species attract attentions owing to their scents, medicinal properties, and ambiguous relationship in the phylogenetic tree. Here, we report a high-quality genome assembly of *Cinnamomum camphora*, based on which two whole-genome duplication (WGD) events were detected in the *C. camphora* genome: one was shared with Magnoliales, and the other was unique to Lauraceae. Phylogenetic analyses illustrated that Lauraceae species formed a compact sister clade to the eudicots. We then performed whole-genome resequencing on 24 *Cinnamomum* species native to China, and the results showed that the topology of *Cinnamomum* species was not entirely consistent with morphological classification. The rise and molecular basis of chemodiversity in *Cinnamomum* were also fascinating issues. In this study, six chemotypes were classified and six main terpenoids were identified as major contributors of chemodiversity in *C. camphora* by the principal component analysis. Through *in vitro* assays and subcellular localization analyses, we identified two key terpene synthase (TPS) genes (*CcTPS16* and *CcTPS54*), the products of which were characterized to catalyze the biosynthesis of two uppermost volatiles (i.e. 1,8-cineole and (iso)nerolidol), respectively, and meditate the generation of two chemotypes by transcriptional regulation and compartmentalization. Additionally, the pathway of medium-chain triglyceride (MCT) biosynthesis in Lauraceae was investigated for the first time. Synteny analysis suggested that the divergent synthesis of MCT and long-chain triglyceride (LCT) in Lauraceae kernels was probably controlled by specific medium-chain fatty acyl-ACP thioesterase (FatB), type-B lysophosphatidic acid acyltransferase (type-B LPAAT), and diacylglycerol acyltransferase 2b (DGAT 2b) isoforms during co-evolution with retentions or deletions in the genome.

## Introduction

Lauraceae is one of the seven families of Laurales that were classified into 45 genera. Laurales together with Magnoliales, Canellales, and Piperales constitute the magnoliids, which includes about 9000 species [1]. According to a few previous studies of Lauraceae and other Mesangiospermae genomes [2–10], the phylogenetic position of magnoliids remained uncertain. Therefore, more genomic data from Lauraceae would be useful for resolving the relationships within angiosperms [11]. In Lauraceae, the genus *Cinnamomum*, more specifically, the Asian *Cinnamomum*, includes approximately 250 species that are widely distributed in the subtropical and tropical regions of Asia. These species were previously divided into sect. *Camphora* and sect. *Cinnamomum* on the basis of morphological characteristics. However, the genetic relationship among these species is undetermined due to some ambiguous morphological traits [12, 13]. Furthermore, the phylogenetic topologies obtained based on chloroplast DNA makers [14], nuclear markers [15], and chloroplast genomes [16] are inconsistent with morphological classification to some extent. Therefore, it is necessary to construct a more reliable species tree using whole-genome data. However, up to now, few genomes are available for *Cinnamomum* species.

*Cinnamomum* species are rich in essential oils, and are thus used as fragrances, spices, and traditional herbs worldwide. For decades, dozens of essential oil profiles and chemotypes have been identified in *Cinnamomum* species, and the primary components (>50%) of essential oils are terpenoids and phenylpropanoids [17]. The promiscuous activities of terpene synthase (TPS), to a large extent, contribute greatly to the tremendous diversity of terpenoid biosynthesis [18], and neofunctionalization, subfunctionalization, and compartmentalization of its paralogs further encourage diversification of terpenoids during evolution [19, 20]. However, the molecular basis of secondary metabolite biosynthesis and chemotype diversity in *Cinnamomum* remains largely unclear. A previous study has reported that the expression level of functional *TPS* genes is pivotal for determining product contents [21], and *trans*-acting factors, such as microRNAs (miRNAs) [22, 23] and transcription factors [24, 25], directly or indirectly affect the expression of *TPS* genes to regulate terpenoid contents *in planta*. Although the expansion of the *TPS* family in the *Cinnamomum kanehirai* [2] and *Litsea cubeba* [9] genomes partially accounts for the diversification of terpenoids in Lauraceae, few key members that produce main components have been isolated in the laurel family [26–28], and the regulatory mechanisms involved in terpenoid contents and chemodiversity are also veiled. Therefore, identifying the key genes and regulatory mechanisms involved in the biosynthesis of primary components of essential oils would shed light on the generation of chemodiversity in *Cinnamomum*.

Lauraceae plants are also considered as new sources of biodiesel due to significant production of kernel oil from their abundant fruits. Among them, seeds of genera *Cinnamomum*, *Litsea*, *Lindera*, *Actinodaphne*, *Umbellulana*, *Neolitsea*, *Sassafras*, and *Laurus* all contain high levels of medium-chain triglyceride (MCT). Instead, plants of the other Lauraceae genera, such as *Persea*, *Phoebe*, *Machilus*, *Neocinnamomum*, and *Cryptocarya*, all exclusively accumulate long-chain triglyceride (LCT) in their kernels. At present, the distinctive mechanisms responsible for divergent biosynthesis of triglycerides in the kernels of Lauraceae plants remain a mystery. Putative proteins involved in LCT formation have been identified from seed transcriptomes of *Neocinnamomum caudatum* [29] and *Persea americana* [30, 31]. Whereas, except for specific medium-chain fatty acyl-ACP thioesterase (Fat) B, which was firstly isolated from *Umbellulana californica* (UcFatB1) [32,33], the biosynthetic pathway of MCT is covered in the laurel family*.* UcFatB1 and its orthologs are ubiquitous in MCT-producing plants and are acknowledged as key regulators to determine chain length of medium-chain fatty acid (MCFA) [34, 35]. In an *in vitro* assay, UcFatB1 was observed to hydrolyze the growing acyl thioesters prematurely to yield C12:0 [33]. When ectopically expressed in oil crops, UcFatB1 reconstituted the fatty acid profiles of seed oils to produce an amount of MCFAs [36,37]. Other proteins from *Cuphea* or Cocoeae, including specific ketoacyl-ACP synthase (KAS) IV [38] and acyl carrier protein (ACP) 2 [39], which function in *de novo* MCFA synthesis, and preferential lysophosphatidic acid acyltransferase (LPAAT) [40, 41] and diacylglycerol acyltransferase (DGAT) [42, 43], which are involved in MCT assembly, demonstrate additive effects on UcFatB1 to promote MCT accumulation in modified oil crops [39, 40]. However, the orthologs of these proteins as well as their functions related to MCT biosynthesis in Lauraceae have not been reported and require further clarification.

Camphor tree (*Cinnamomum camphora* (L.) Presl.) is widely cultivated in South China for its economic and ecological importance. Its leaf essential oils (LEOs) contain dozens of terpenoids [44], which have important industrial and pharmaceutical applications. For example, linalool is one kind of famous perfume compound and exerts antiproliferative activity against various cancer cells [45]; (+)-borneol is used as permeation in enhancing drug delivery across various physiological barriers [46]. Key *TPS* genes involved in biosynthesis of main terpenoids have not been identified in camphor tree. Structural similarity predictions indicate that camphor and (+)-borneol probably arose from the same carbocation and are products of the same TPS. Recently, three borneol dehydrogenases (CcBDH1, CcBDH2, and CcBDH3) from camphor tree were characterized to catalyze (+)-borneol into camphor *in vitro* [47]_._ The seed of camphor tree can accumulate a high level of MCT [48], and the annual output of camphor tree seeds was estimated to be 10 million tons with great potential for functional and edible oils in China. In summary, due to a lack of genomic data, the knowledge about LEOs, storage lipids, and genetic improvement of *C. camphora* is limited.

Here, we report a chromosome-level genome assembly of *C. camphora*. Based on this genome assembly and in combination with whole-genome resequencing data of 23 other *Cinnamomum* species, we constructed phylogenetic trees for *Cinnamomum* species. We sought to explore the genetic relationship of these species within the *Cinnamomum* genus and to clarify the phylogenetic position of the magnoliids, which are both undetermined. With the assistance of multi-omics data and biochemical analyses, a comprehensive, genome-wide analysis of the TPS family was applied to characterize the key members and the regulatory mechanisms involved in the biosynthesis of five main terpenoids and formation of chemodiversity. In addition, the genomes of *C. camphora* and other four Lauraceae species [2, 5, 6, 9] provided access to genes related to MCT biosynthesis and an understanding of the heterogeneity in the laurel seeds in response to divergent biosynthesis of triglycerides. Overall, our findings provide insights into genetic relationships, chemodiversity, and MCT biosynthesis in *Cinnamomum*, as well as a molecular basis for breeding high-terpenoid-content varieties.

## Results

### Genome sequencing, assembly, and annotation

**Figure 1:**
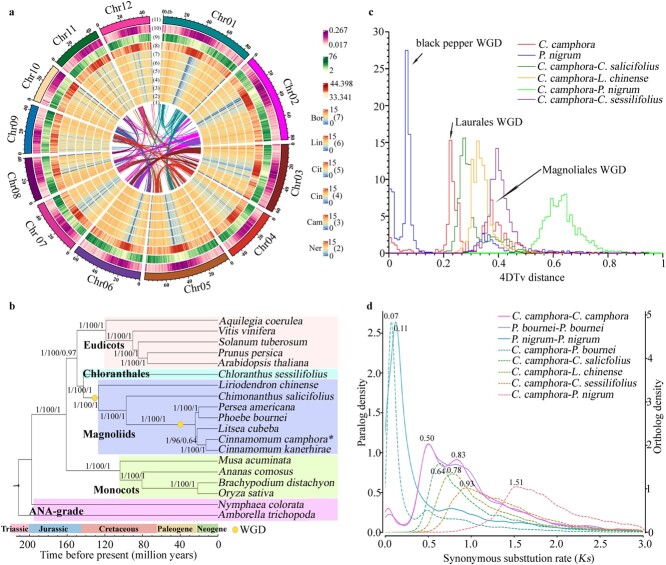
Landscape, phylogeny, and WGDs of the *C. camphora* genome. **a** Circos plot of the *C. camphora* genome. Circles from the outside inwards: (11) pseudochromosomes, (10) repeat density, (9) gene density, (8) GC content, (7 to 2) FPKM values of genes in the leaf transcriptome of the six chemotypes, (1) identified syntenic blocks. **b** Timescale and phylogeny reconstructed on the basis of the low-copy nuclear (LCN) and single-copy orthologous genes using concatenation and multi-species coalescent (MSC)-based approaches. Posterior probabilities (PPs) and bootstrap (BS) support values are indicated at each internal branch, slash-separated from left to right, are: (i) PP of a coalescent species tree on the basis of LCN genes using ASTRAL; (ii) BS value of a concatenated super-matrix species tree on the basis of single-copy orthologous genes using IQ-TREE; and (iii) PP of a coalescent species tree on the basis of single-copy orthologous genes using ASTRAL. **c** Distribution of the 4DTv distance between duplicated genes in syntenic blocks within *C. camphora* as well as between *C. camphora* and other species. **d***Ks* distribution. *Ks* values within a genome are indicated by solid lines, and a peak represents one WGD event. *Ks* values among species are indicated by dashed lines, and a peak represents a divergence event.

The *k*-mer analysis revealed that the genome size of *C. camphora* was 723.12 Mb with a heterozygosity rate of 1.24% and a repetitive sequence content of 46.69% (Fig. S1, see online supplementary material). We applied a combination of three sequencing technologies to obtain a chromosome-level genome assembly of *C. camphora*: Illumina sequencing (generated 109.76 Gb raw data), PacBio sequencing [10 single molecule real time (SMRT) cells yielded a total of 77.79 Gb raw data], and high-throughput chromosome conformation capture (Hi-C) sequencing (produced 80.55 Gb raw data) (Tables S1–S3, see online supplementary material). Approximately 78.62 Mb of redundant contigs representing heterozygous regions were removed, and the polished contigs were 705.97 Mb in size with an N50 of 2.19 Mb, while the constructed scaffolds were 706.47 Mb in size with an N50 of 3.17 Mb (Table S4, see online supplementary material). Finally, by virtue of Hi-C sequencing data, 703.92 Mb of the assembled sequences were anchored onto 12 pseudochromosomes (Chr), and 97.85% of the sequences were directional and in order (Fig. 1a; Fig. S2; Tables S5 and S6, see online supplementary material).

Bacterial artificial chromosome (BAC) clones, RNA-sequencing (RNA-seq) unigenes, and Benchmarking Universal Single-Copy Ortholog (BUSCO) analysis were utilized to assess the quality and completeness of the genome assembly. The coverage rate obtained from five BAC clones and RNA-seq unigenes was 88.58 ~ 99.83% and 98.20%, respectively, and the completeness rate was 95.20% by BUSCO assessment (Fig. S3; Tables S7–S11, see online supplementary material), suggesting the *C. camphora* genome assembly was high-quality and suitable for subsequent analysis.

Using a combination of homology-based and *de novo* prediction approaches, 59.64% of the genome was identified as non-redundant repeat elements, and 36 411 protein-coding genes were annotated (Figs. 4 and S5; Tables S12–S14, see online supplementary material). These genes, on average, contained 5.05 exons and were 1585.56 bp in length of coding sequences. About 95.44% of these genes were supported by mRNA, homology-based, and/or *de novo* predictions, with >50% coverage (Fig. S6; Tables S14 and S15, see online supplementary material), and BUSCO analysis also showed that 90% of them were intact (Table S16, see online supplementary material). Following that, 30 117 genes were assigned to entries in seven functional databases, including NR, SWISS-PROT, KEGG, KOG, TrEMBL, InterPro, and GO, and 19 729 genes were shared by the NR, InterPro, KEGG, SWISS-PROT, and KOG databases (Fig. S7; Table S17, see online supplementary material).

### Phylogenetic analysis and whole-genome duplication (WGD) events

The high-quality *C. camphora* genome allowed exploration of the aforementioned discordance in the phylogenetic position of the magnoliids within angiosperms. According to the 214 single-copy orthologous genes identified from the genomes of 19 species, including five eudicots, four monocots, seven magnoliids, one Chloranthales species, and two ANA-grade species, high-confidence phylogenetic trees were constructed by concatenation and multi-species coalescent (MSC)-based approaches (Table S18, see online supplementary material). The phylogenetic tree constructed based on the concatenation method revealed that the magnoliids and Chloranthales fell into one clade sister to the eudicots rather than the monocots (Fig. 1b). Given that incomplete lineage sorting might compromise the resolution of early-diverging branches within angiosperms, the MSC-based phylogenetic analyses inferred by ASTRAL, using each gene tree from the 214 single-copy orthologous genes, were also applied separately and produced a tree having the same topology with high posterior probabilities (Fig. 1b; Fig. S8, see online supplementary material). Furthermore, to reduce the influence of methodological orthology inference, 1260 low-copy nuclear (LCN) genes (single-copy in at least 17 of the 19 species, with a maximum of 22 genes allowed in total) were identified in all 19 species to construct a phylogenetic tree. The LCN-based phylogeny again supported that the magnoliids and Chloranthales formed a sister clade to the eudicots after their common ancestor diverged from the monocots (Fig. 1b; Fig. S9, see online supplementary material).

In addition, the divergence time of Lauraceae from *Chimonanthus salicifolius* was estimated to be 97.4 million years ago (Mya), and the divergence time between *C. camphora* and *C. kanehirae* was 12.5 Mya (Fig. S10; Table S19, see online supplementary material). We then analysed the expansion and contraction of 12 047 common gene families shared among the 19 species (Fig. S11, see online supplementary material). In *C. camphora*, there were 183 significantly expanded families (*P*-value <0.01), consisting of 2112 genes enriched in 11 KEGG pathways involved in plant-pathogen interaction and secondary metabolite biosynthesis (Table S20, see online supplementary material).

In the investigation of genome collinearity and paralog age distribution, the two peaks observed from estimation of fourfold degenerate site transversion (4DTv) distribution indicated that *C. camphora* experienced two rounds of WGD (Fig. 1c). The recent peak (about 0.22) was smaller while the ancient peak (about 0.37) was greater than those of *C. camphora*-*C. salicifolius* and *C. camphora*-*Liriodendron chinense*, indicating that the ancient WGD event occurred in a common ancestor of Laurales and Magnoliales, while the recent one occurred after the divergence of Magnoliales from Laurales. The peak of *C. camphora*-*Piper nigrum* (about 0.64) was larger than the ancient peak (about 0.37) showing that the ancient WGD event occurred in Laurales genomes after the differentiation of Laurales and Piperales. The synonymous substitution rate (*Ks*) of gene pairs in syntenic blocks was then implemented to examine the two polyploidization events in the *C. camphora* genome. The *Ks* distribution showed two peaks in Lauraceae (*C. camphora* and *Phoebe bournei*) genomes, with values of around 0.50 and 0.83, respectively, which confirmed that the same polyploidization events were shared among the laurel family (Fig. 1d). The ancient *Ks* peak was smaller than the differentiation peaks of *C. camphora-Chloranthales sessilifolius* (*Ks* ≈ 0.93) and *C. camphora*-*P. nigrum* (*Ks* ≈ 1.51), but was larger than peaks indicating the divergence of *C. camphora* and *C. salicifolius* (*Ks* ≈ 0.64), and *C. camphora* and *L. chinense* (*Ks* ≈ 0.78), respectively, implying that the ancient polyploidization event occurred in the common ancestor of Laurales and Magnoliales. On the contrary, the recent *Ks* peak was smaller than the peak of *C. camphora*-*C. salicifolius* (*Ks* ≈ 0.64), which indicated that the recent polyploidization event was later than the divergence between Lauraceae and Calycanthaceae and independently occurred in the laurel family, in accordance with the results from the 4DTv analysis.

### Phylogenetic relationships of *Cinnamomum* species revealed by whole-genome resequencing

There were 47 species and one variety of the Asian *Cinnamomum* native to China. Based on the *C. camphora* genome, we re-sequenced the whole genomes of 42 individuals from 24 species to explore their relationships and generate 372.02 GB of data (Table S21, see online supplementary material). A total of 53.37 million single nucleotide polymorphisms (SNPs) and 2.67 million small insertions and deletions (InDels) were obtained when compared to the *C. camphora* genome (Table 1). Sect *Cinnamomum* had all higher ratios of transversion to transition of SNPs, homozygous to heterozygous of SNPs, and homozygous to heterozygous of small InDels than these of sect *Camphora,* correspondingly, which indicated that *C. camphora* was closer to other species of sect *Camphora* than to species of sect *Cinnamomum* at the genome level (Table S21, see online supplementary material).

**Table 1:** Identification of SNPs and small inDels in *Cinnamomum* by whole genome resequencing.

**Species**	**Clean_reads (million)**	**Clean_base (GB)**	**Q20 (%)**	**GC (%)**	**Map (%)**	**Ave_depth**	**SNPs(million)**	**InDels in CDSs (thousand)**	**InDels in genome (million)**	**SNPs in genes**	**InDels in genes**
*C. tamala*	27.59	8.26	96.82	38.69	89.35	6	1.31	13.34	0.72	20 589	8714
*C. wilsonii*	30.30	9.06	96.99	40.09	88.37	6	1.28	13.04	0.68	20 672	8649
*C*. *kotoense*	26.52	7.90	97.53	40.09	90.27	5	1.14	11.77	0.59	20 047	8031
*C. appelianum*	29.39	8.80	97.62	39.35	95.50	9	1.43	8.23	0.65	18 808	5678
*C.bejolghota*	27.52	8.24	97.49	40.87	86.09	5	1.07	10.22	0.54	19 418	7275
*C. iners*	26.47	7.92	97.75	40.27	89.96	6	1.21	12.42	0.64	20 298	8328
*C. cassia*	26.68	7.95	97.57	39.93	88.62	8	1.22	12.60	0.53	20 234	8338
*C. pauciflorum*	29.55	8.85	97.29	40.23	88.03	6	1.19	11.41	0.65	19 947	7847
*C. osmophloeum*	29.17	8.73	97.60	39.95	88.37	6	1.24	12.53	0.66	20 376	8357
*C. japonicum*	26.95	8.07	97.61	39.57	91.22	6	1.18	12.25	0.63	20 101	8163
*C. subavenium*	27.20	8.14	97.60	39.53	89.52	5	1.09	10.48	0.56	19 404	7386
*C. zeylanicum*	28.64	8.56	97.00	39.92	87.53	5	1.17	10.96	0.59	20 022	7681
*C. heyneanum*	27.29	8.18	97.29	39.69	90.28	6	1.18	11.71	0.63	19 873	7972
*C. burmanni*	27.56	8.26	97.25	39.31	90.72	6	1.21	12.40	0.65	20 128	8277
*C. chartophyllum*	27.48	8.21	97.29	39.12	96.04	9	1.35	8.18	0.62	18 784	5539
*C. micranthum*	29.83	8.93	96.95	39.25	94.18	9	1.30	7.51	0.57	18 460	5216
*C. bodinieri* Cin-type	27.82	8.33	97.04	39.09	96.89	8	1.57	8.72	0.68	19 325	6037
*C. bodinieri* Lin-type	28.94	8.66	97.27	38.77	96.80	9	1.50	8.81	0.68	19 364	6111
*C. bodinieri* Cam-type	29.16	8.73	97.22	39.09	94.55	8	1.53	9.08	0.70	19 545	6283
*C. bodinieri* Cit-type	27.45	8.22	97.21	39.13	95.35	8	1.52	8.64	0.67	19 320	5997
*C. bodinieri* Ner-type	28.79	8.62	97.00	40.69	86.23	4	1.06	10.10	0.52	19 213	7099
*C. parthenoxylon* Lin-type	29.37	8.79	97.36	38.08	97.72	9	1.49	8.47	0.69	19 045	5871
*C. parthenoxylon* Bor-type	27.74	8.31	97.11	38.69	97.67	9	1.49	8.62	0.69	19 194	5910
*C.parthenoxylon* Cam-type	27.78	8.32	97.36	39.10	96.68	9	1.42	8.25	0.65	18 802	5722
*C. parthenoxylon* Cit-type	28.52	8.54	97.33	38.97	96.05	8	1.45	8.33	0.67	18 890	5795
*C. parthenoxylon* Ner-type	29.39	8.80	97.62	39.35	95.50	9	1.43	8.23	0.65	18 808	5678
*C. parthenoxylon* Cin-type	27.72	8.25	97.50	38.75	96.42	8	1.39	7.94	0.63	18 721	5574
*C. tenuipilum*	28.49	8.53	97.21	39.76	93.90	9	1.30	7.93	0.59	18 508	5394
*C. mollifolium*	27.09	8.10	97.57	39.93	88.62	8	1.22	7.01	0.60	17 932	4941
*C. septentrionale* Lin-type	27.57	8.25	97.51	39.12	96.07	9	1.41	8.17	0.62	18 409	5712
*C. septentrionale* Cam-type	27.69	8.29	97.26	38.78	97.03	8	1.26	6.95	0.55	16 818	4918
*C. septentrionale* Cit-type	27.37	8.20	97.23	38.75	97.08	8	1.52	8.57	0.67	19 298	5929
*C. septentrionale* Cin-type	29.47	8.83	97.31	39.33	95.43	8	1.30	7.63	0.59	17 308	5327
*C. septentrionale* Ner-type	27.80	8.33	97.39	39.08	96.48	8	1.14	6.36	0.49	15 744	4551
*C. glanduliferum*	28.53	8.50	97.54	40.30	89.37	9	1.28	7.56	0.57	18 285	5228
*C. longipaniculatum*	27.12	8.12	96.85	38.92	94.91	7	1.39	7.56	0.60	18 519	5347
*C. camphora* Cam-type	34.36	10.29	98.05	38.68	95.98	10	0.92	5.25	0.41	12 749	3736
*C. camphora* Bor-type	42.15	12.63	98.00	38.42	97.27	14	1.01	5.76	0.44	13 437	4031
*C. camphora* Ner-type	36.73	11.01	98.06	38.69	97.16	12	0.94	5.33	0.41	12 759	3759
*C. camphora* Cin-type	37.89	11.35	98.06	39.09	96.62	12	0.95	5.51	0.42	13 123	3845
*C. camphora* Lin-type	37.59	11.26	98.05	38.94	96.98	12	1.30	5.39	0.42	12 822	3809
*C. camphora* Cit-type	40.56	12.15	98.12	39.08	96.33	12	0.94	7.31	0.57	16 503	5115

**Figure 2:**
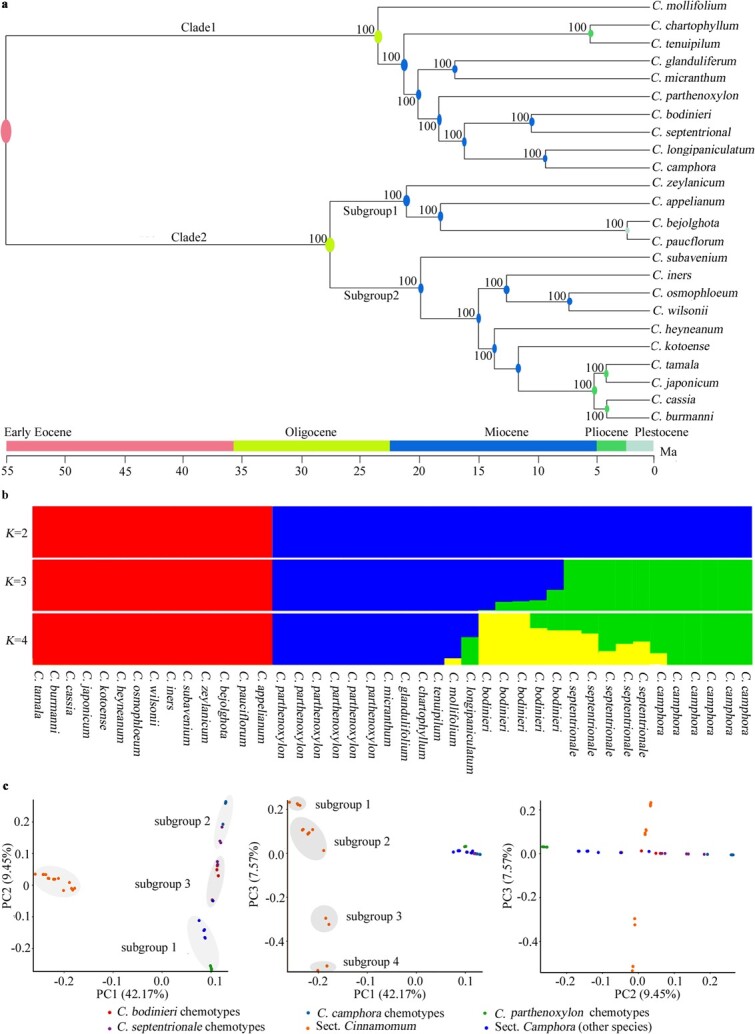
Phylogenetic relationship of the Asian *Cinnamomum* species native to China *via* whole-genome resequencing. **a**. Phylogenetic relationships of the Asian *Cinnamomum* species native to China. The 24 *Cinnamomum* species were separated into sect. *Camphora* (Clade 1) and sect. *Cinnamomum* (Clade 2) according to non-random missing SNP sites. Chronogram of Asian *Cinnamomum* speciation was estimated, during Oligocene to Plestocene. Bayesian posterior probability is 1.0 for all nodes. **b** Structural analysis of 42 individuals belonging to the 24 *Cinnamomum* species. At *K* = 4, one ancestor and three additional ancestors were traced from sect. *Cinnamomum* and sect. *Camphora*, respectively. **c** PCA of the 42 individuals, yielding three and four subgroups in sect. *Camphora* and sect. *Cinnamomum*, respectively.

With non-random missing SNP sites in all species, an unrooted phylogenetic tree was constructed, yielding two clades (Fig. 2a; Fig. S12, see online supplementary material). Clade 1 contained all sect. *Camphora* species, and a closer relationship was found between *Cinnamomum longipaniculatum* and *C. camphora.* The 14 species of sect. *Cinnamomum* fell into clade 2 and two subgroups were also identified, including four species and 10 species, respectively. Referring to origin of the *Cinnamomum* group (about 55 Ma) [15], estimation of speciation times of the 24 Asian *Cinnamomum* species were performed, from Oligocene to Pleistocene (Fig. 2a).

In the structure analysis, the population stratification was assessed on the basis of *K* values (from 1 to 10) of the log likelihood of data (Fig. 2b). At *K* = 4, the cross-validation (cv) error was lowest (Fig. S13, see online supplementary material). The group, consisting of 14 species of sect. *Cinnamomum*, shared a common ancestor. However, three additional ancestors were traced from sect. *Camphora* and were shared by *C. camphora*, *Cinnamomum bodinieri*, and a third subgroup including *Cinnamomum tenuipilum*, *Cinnamomum chartophyllum*, and *Cinnamomum micranthum*. *C. longipaniculatum*, *Cinnamomum mollifolium*, and *Cinnamomum septentrionale* appeared to originate from hybridization between *C. camphora* and the third ancestor, *C. bodinieri* and the third ancestor, and *C. bodinieri* and *C. camphora,* respectively*.* Furthermore, a principal component analysis (PCA) showed that principal component 1 (PC1; 42.17%) played a leading role in the divergence of sect. *Camphora* from sect. *Cinnamomum* (Fig. 2c)*.* Additionally, the sect. *Camphora* species segregated along PC2, whereas the sect. *Cinnamomum* species segregated along PC3. Three subgroups were found in sect. *Camphora*, which was consistent with the genetic structure (*K* = 4). In sect. *Cinnamomum*, four subgroups were also observed. In addition, the inconsistent chemotype topographies in *C. bodinieri*, *C. camphora*, *Cinnamomum parthenoxylon*, and *C. septentrionale* all suggested that random selection facilitated chemodiversity in *Cinnamomum* species (Fig. S14, see online supplementary material).

### Genome-wide analysis of the *TPS* gene family and identification of key candidates responsible for the biosynthesis of main terpenoids

Commonly, according to the uppermost terpenoids of LEOs, camphor trees are empirically classified into five chemotypes, including linalool (Lin)-, (iso) nerolidol (Ner)-, 1,8-cineole (Cin)-, camphor (Cam)-, and (+)-borneol (Bor)-types [44]. In this study, LEO profiles from a population of 250 individuals collected in Jiangxi Province of China were investigated. Four monoterpenoids, including camphor (34.35–77.14%), 1,8-cineole (25.11–62.80%), linalool (73.45–91.95%), and (+)-borneol (26.94–77.32%), and one sesquiterpenoid, *i.e.* (iso) nerolidol (30.75–43.43%), were identified as uppermost components, respectively (Table S22, see online supplementary material). The top 20 compounds from each individual were subjected to PCA analysis (Table S23, see online supplementary material). The first four principal axes represented 92.34% of the total information, and the 250 samples were divided into five chemotypes, in accordance with the empirical classification (Fig. S15, see online supplementary material). In addition, a rare scent was also found in the leaves of a few other individuals, in which mixtures of *cis-* and *trans*-citrals were the main components [designated as citral (Cit)-type] (Table S22, see online supplementary material).

**Figure 3:**
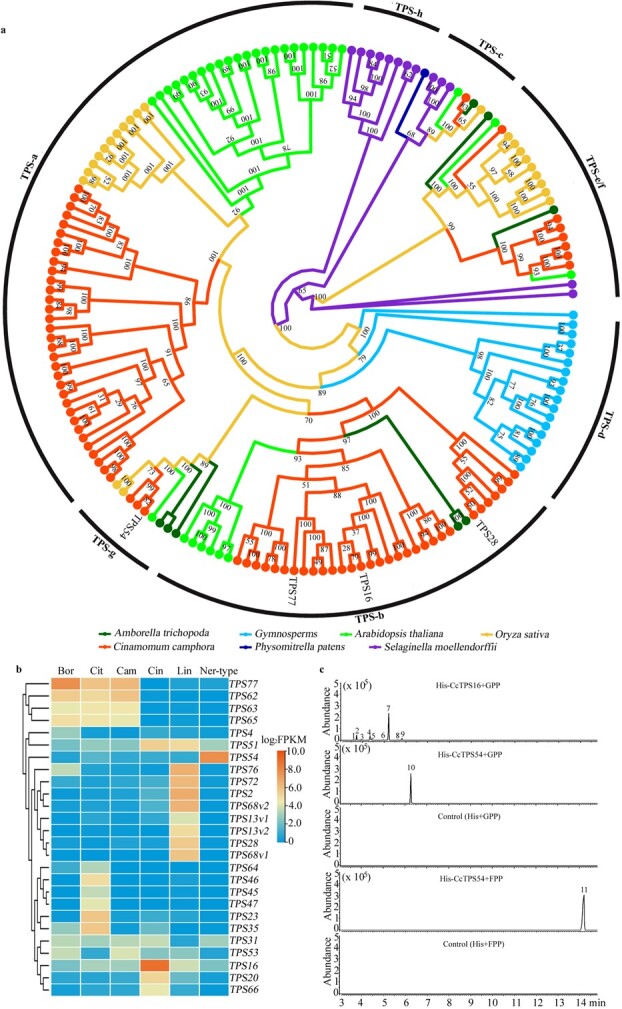
Phylogenetic positions of CcTPSs and candidate genes responsible for the biosynthesis of main terpenoids. **a** Phylogenetic tree of CcTPS genes. Putative full-length TPS proteins identified in *C. camphora* (Data S1, see online supplementary material) and six other sequenced plant genomes (>200 amino acids in length) were subjected to phylogenetic analysis. The TPS subfamilies are shown along the circumference of the circle. **b** Clustering of deferentially expressed *CcTPS* genes among the six chemotypes according to the FPKM values from the leaf transcriptomes. **c***In vitro* characterization of recombinant CcTPSs. The recombined enzyme expressed in *E. coli* BL21 (DE3) Rosetta cells was purified by Ni^2+^ affinity. With GPP and FPP as substrates, respectively, the products of recombinant TPSs were further identified by Gas Chromatography-Mass Spectrometer (GC-MS) analysis. 1, *α*-thujene; 2, *α*-pinene; 3, camphene; 4, *β*-pinene; 5, *β*-myrcene; 6, *α*-terpinene; 7, 1,8-cineole; 8, *γ*-terpinene; 9, *α*-terpinolene; 10, linalool; 11, (iso)nerolidol.

TPSs were the key enforcers in terpenoid biosynthesis. To identify key *TPS* genes responding to the biosynthesis of main terpenoids, the full-length *CcTPS* genes were firstly obtained by scanning the *C. camphora* genome (Figs. S16 and S17; Table S24, see online supplementary material). A total of 78 *CcTPS* genes were recognized and divided into six subfamilies based on phylogenetic analyses, which were labeled CcTPS-a to CcTPS-g without CcTPS-d, as described previously [19] (Fig. 3a). Remarkably, the CcTPS-b subfamily, which encodes angiosperm-specific monoterpene synthases, was substantially expanded with 32 members, while the CcTPS-g subfamily having only three members encoding synthases for acyclic terpenoids of floral scent. Members from the CcTPS-b and CcTPS-g subfamilies were expected to facilitate formation of the six main terpenoids. Chromosomal localization further uncovered that *CcTPS* genes were unequally distributed throughout 11 chromosomes except for Chr12 (Fig. S18, see online supplementary material). The two largest clusters, containing 27 and 32 *TPS* genes, were located on Chr09 and Chr11, respectively (Fig. S19a–c; Table S24, see online supplementary material). Specifically, in the 16.24–22.05 Mb interval of Chr11, some paralogs from both the CcTPS-a and CcTPS-b subfamilies arranged in a tandem array and showed mosaic distribution patterns (Fig. S19a–c, see online supplementary material), suggesting that tandem duplication and segmental duplication events both significantly promoted the expansion of the *TPS* gene family in the *C. camphora* genome.

Eighteen leaf transcriptomes (in biological triplicate) of the six chemotypes were obtained to further assist in identifying key *CcTPS*s involved in the biosynthesis of main terpenoids (Fig. S20a; Table S25, see online supplementary material). Differently expressed genes (DEGs) related to terpenoid biosynthesis were given attention by annotation and enrichment analyses. Based on the fragments per kilobase of transcript per million fragments (FPKM) values, the differently expressed *CcTPS*s, which fell into the TPS-b and TPS-g subfamilies, respectively, were found among chemotypes (Fig. 3b; Fig. S20b, see online supplementary material). The nucleotide sequence similarities within genes from group1 and 2 in the TPS-b subfamily were both over 98%. *CcTPS16*, *CcTPS28*, and *CcTPS54* with significantly higher expression in Cin-, Lin-, and Ner-type, respectively, were verified by quantitative real-time polymerase chain reaction (qRT-PCR) analysis (Fig. S21, see online supplementary material) and were identified as the candidate gene coding for 1,8-cineole synthase, linalool synthase, and (iso) nerolidol synthase according to cDNA clone, correspondingly. *CcTPS77* with significantly higher expression in Cit-, Cam-, and Bor-types was identified as a candidate gene involved in (+)-borneol (camphor as its dehydrogenation product) or citral biosynthesis (Fig. 3b). Further, comparisons of LEO profiles among samples were performed to infer biological function of CcTPS77. The results revealed that camphor was not only one of the uppermost components in Cam-type but also significantly accumulated in Cit-type (Table S22, see online supplementary material), implying that CcTPS77 might be a borneol synthetase rather than a citral synthetase. Three additional samples from Cit-type, which had a low level of camphor (Table S22, see online supplementary material), were employed to verify the expression level of *CcTPS77* among the six chemotypes by qRT-PCR. As expected, *CcTPS77* was up-regulated in both Cam- and Bor-types but with low expression levels in these three additional samples (Fig. S21, see online supplementary material). In addition, only *CcBHD1* exhibited a higher expression level in the leaf of Cam-type relative to that of Bor-type by qRT-PCR analysis (Fig. S21, see online supplementary material).

Subsequently, the recombinant proteins of CcTPS16 and CcTPS54 with His-tag were harvested in *Escherichia coli* BL21 (DE3) cells. With geranyl pyrophosphate (GPP) as a substrate, the product of CcTPS54 was linalool, but that of CcTPS16 included substantial 1,8-cineole, *α*- and *β*-pinene, *α*-terpinolene, and a few other monoterpenes (Fig. 3c; Fig. S22, see online supplementary material). Contrastingly, with farnesyl pyrophosphate (FPP) as a substrate, the product of CcTPS54 was (iso) nerolidol, and no detectable product of CcTPS16 was formed (Fig. 3c; Fig. S22, see online supplementary material). CcTPS16 was unmistakably identified as 1,8-cineole synthase on the basis of the results from the above-mentioned expression and enzyme catalysis analyses. However, given the much higher expression in Ner-type than in Lin-type, the bi-functional CcTPS54 should be endowed as (iso) nerolidol synthase rather than linalool synthase *in vivo*. Plant cell compartmentation separated the plastidal monoterpene biosynthesis from the cytosolic/peroxisomal sesquiterpenoid biosynthesis [49]. Therefore, the subcellular localization of CcTPS54 was crucial to its physiological function to synthesize (iso) nerolidol or linalool *in planta*. To reveal the subcellular localization of CcTPS16 and CcTPS54, recombinant vectors containing full-length CcTPS16-GFP (green fluorescent protein) and CcTPS54-GFP fusion proteins were transiently expressed in *Nicotiana benthamiana* leaves. Confocal scanning microscopic analysis showed that CcTPS16 was localized in the plastid, while CcTPS54 resided in the cytosol, confirming the involvement of CcTPS54 in (iso) nerolidol biosynthesis *in vivo* (Fig. S23, see online supplementary material).

Furthermore, we investigated miRNAs and their target genes related to terpenoid biosynthesis in the leaves of five chemotypes (Lin-, Cin-, Bor-, Cam-, and Ner-type) (Tables S26–S29, see online supplementary material). A total of 28 miRNAs were predicted to probably target genes involved in the methylerythritol phosphate and mevalonic acid pathways, or coding for the *trans*-isopentenyl diphosphate synthases and TPSs (Figs S24 and S25a; Tables S30–S32, see online supplementary material). Of them, miR4250, miR8717, miR159a-3p, and cam-mir-618 targeted *CcTPS16*, *CcTPS28*, *CcTPS54*, and *CcTPS77*, respectively, and showed differential expression among the five chemotypes (Fig. S25b; Tables S30 and S33, see online supplementary material). Concretely, miR159a-3p was significantly up-regulated in Ner-type and probably acted as an important regulator of (iso) nerolidol biosynthesis by a feedback mechanism, whereas miR4250 was highly accumulated in Lin-type and might play a role in the negative regulation of 1,8-cineole content in Lin-type.

### Genetic basis of Lauraceae responding to divergent triglyceride biosynthesis

The predominant triglyceride species in the seed of camphor tree were C12:0/10:0/10:0 and C10:0/12:0/10:0 [48]. To understand the molecular basis of MCT biosynthesis in camphor tree, a set of genes involved in *de novo* fatty acid synthesis, trafficking, and triglyceride assembly and coalescence were investigated and identified in the *C. camphora* genome according to homologs from *Arabidopsis* and other oilseed plants (Table S34, see online supplementary material). Of them, specific orthologs of KASs, ACPs, FATs, LPAATs, and DGATs that have been documented with preferential molecular functions in other MCT-producing plants were extensively inspected (Table S34, see online supplementary material).

Specifically, two KAS isoforms were clustered in the KAS II/IV subgroup, but whether they were orthologs to KAS IV [38] remained unclear because high sequence similarity was observed between KAS II and KAS IV, and KAS II/IV evolved independently in *Cupeha* (Fig. S26a, see online supplementary material). Similarly, ACP isoforms from Lauraceae, Cocoeae, and *Cuphea* formed independent branches respectively, and it was also difficult to decide whether ACPs in camphor tree had a similar preference to ClACP2 [39] for MCFA production (Fig. S26b, see online supplementary material). Two FatA isoforms and four FatB isoforms were also found in the *C. camphora* genome (Figs S26c and S27, see online supplementary material). Although CcFatB1 (Genbank accession number AAC49151) was specific to C14:0-ACP in both *in vitro* [50] and ectopic expression [51], no other ortholog of UcFatB1 except itself, with 92% amino acid identity, was identified by the phylogenetic analysis (Fig. S27, see online supplementary material). With regard to MCT assembly, both type-A LPAAT [40] and type-B LPAAT [41] were reported to mediate the MCFA insertion at the *sn*-2 position of triglyceride; therefore, it was indistinguishable that which one of the two type-A and one type-B LPAAT isoforms was responsive to assemble MCT in camphor tree (Fig. S28a, see online supplementary material). By contrast, the only CcDGAT1 isoform was probably the candidate to induce MCT accumulation in camphor tree seeds in light of its orthologs from both *Cuphea pulcherrima* [42] and *Elaeis guineensis* [43] having dominant activities to promote the MCFA insertion at the *sn*-3 position of triglyceride (Fig. S28b, see online supplementary material).

**Figure 4:**
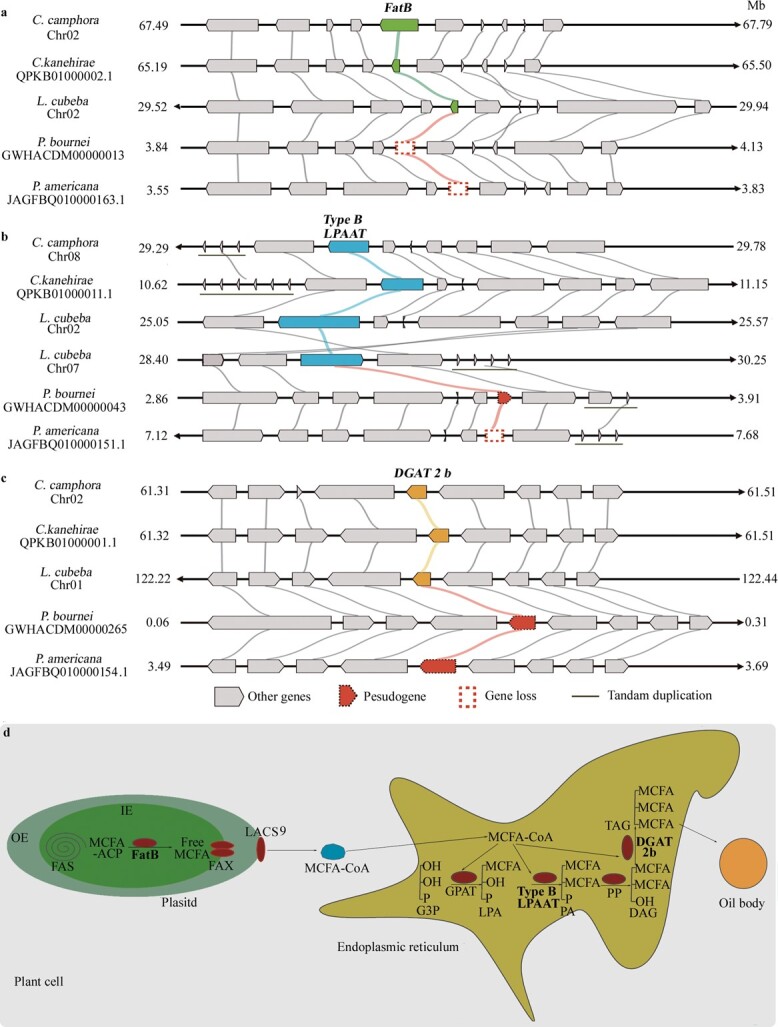
Identification of candidate genes responding to divergent biosynthesis of triglycerides in the laurel family by synteny analyses. **a** The gene (green) coding for medium-chain fatty acyl-ACP thioesterase (an ortholog of UcFatB1) was lost in LCT-producing genomes. **b** The gene (cyan) coding for type-B LPAAT was pseudogenized or lost in LCT-producing genomes. **c** The gene (yellow) coding for DGAT 2b was pseudogenized in LCT-producing genomes. **d** MCTs biosynthesis pathway in MCT-producing Lauraceae species. FAS, fatty acid synthase; FAX, fatty acid export; LACS9, long-chain acyl-CoA synthetase 9; G3P, glycerol 3-phosphate; LAP, lysophosphatidic acid; PA, phosphatidic acid; DAG, diacylglycerol; OE, outer membrane; IE, inner membrane.

Importantly, these candidate proteins were carefully compared between the two LCT-producing genomes (*P. americana* [5] and *P. bournei* [6]) and the other two MCT-producing genomes (*L. cubeba* [17] and *C. kanehirai* [2]) to uncover mechanisms responding to divergent fatty acid biosynthesis in the laurel family. As a result, genes coding for FatA, ACP, KAS, type-A LPAAT, DGAT1, and DGAT2a/c isoforms were all conserved, whereas genes coding for the orthologs of UcFatB1, type-B LPAAT, and DGAT2b were all retained in MCT-producing laurel plants but were lost or pseudogenized in LCT-producing laurel plants indicated by syntenic analyses (Fig. 4a–c). To eliminate sequencing errors that might lead to discrepancies in the above-mentioned orthologs between MCT-producing and LCT-producing species, we further examined other genes in the three small syntenic blocks which contained gene coding for UcFatB1, type-B LPAAT, and DGAT2b, respectively. The high conservation and completeness of the nine flanking genes implied that the discrepancies of the three sets of candidate genes resulted from the distinct evolutionary pattern within the Lauraceae family (Fig. 4a–c). Together, these results suggested that the retained ortholog of UcFatB1 presumably conferred crucial activity on MCFA formation, and that the specific acyltransferases (type-B LPAAT and DGAT2b) controlled MCT assembly in MCT-producing laurel seeds (Fig. 4d).

## **D**i**scussion**

In this study, two rounds of independent WGD events were found in the *C. camphora* genome. The ancient WGD event occurred in the common ancestor of Laurales and Magnoliales, and the recent one occurred posterior to the divergence of Magnoliales from Laurales, similar to those observed in other Lauraceae plants [2, 5, 6, 9], which confirmed that the same polyploidization events are shared in the laurel family.

So far, the phylogenetic position of the magnoliids is unascertained and three ambiguous topologies have been established, i.e. a sister group to the eudicots [2, 3], a sister group to the monocots [4], and a sister group to a clade involving the monocots and eudicots [5–8], according to genomic data from Lauraceae and other Mesangiospermae species. The contradiction in the phylogenetic position of the magnoliids is also seen in the Angiosperm Phylogeny Group (APG) system that three distinct phylogenetic positions are indicated by four versions of the APG system (The APG, 1998, 2003, 2009, and 2016). In this study, we used concatenation and coalescent approaches to construct phylogenetic trees on the basis of single-copy orthologous genes and LCN genes, the topology of angiosperms inferred from the *C. camphora* genome supported that the Magnoliids and Chloranthales formed a sister clade to the eudicots. These results are consistent with the phylogeny obtained from previous analyses based on the *C. kanehirae* [2], *C. salicifolius* [10], and *C. sessilifolius* genomes [3], but disagree with that using the *P. bournei* [5] and *L. chinense* [8] genomes. One possible explanation would be that different data sets (e.g. single-copy genes or LCN genes) and methods (e.g. coalescent methods or concatenation methods) were applied to construct phylogenetic trees, which may lead to phylogenetic discordance of the magnoliids [9]. In addition, although chromosome fusion events [4] support the sister relationship between the monocots and magnoliids, a phylogenomic analysis including the *C. sessilifolius* genome strongly rejects this relationship [3]. Therefore, a sister relationship between the magnoliids and eudicots more likely occurred within the angiosperms and is strongly favored by the *C. camphora* genome.

The 24 *Cinnamomum* species native to China were divided into two clusters by using the whole-genome resequencing data, in line with the classification of sect. *Camphora* and sect. *Cinnamomum.* However, within sect. *Camphora* and sect. *Cinnamomum*, the relationships among species were partly inconsistent with morphological traits. For example, conspicuous hairs on both surfaces or the abaxial surface of mature leaves is a main synapomorphy shared among *C. tenuipilum*, *C. septentrionale*, *C. mollifolium*, and *C. bodinier* of sect. *Camphora* [52]; however, these species were not preferentially clustered in the phylogenetic tree (Fig. 2a). These results suggest that some morphological traits are likely dissociated with the genetic relationship among *Cinnamomum* species. In addition, the topology of the phylogenetic tree was also incompatible with that inferred from other molecular phylogy [15, 16] to some extent. These discrepancies highlight the importance of methodological trade-offs to construct genetic relationships of *Cinnamomum* species.

Based on the *K* value (*K* = 4), three ancestors were traced in sect. *Camphora* (Fig. 2b)*.* The relationship is consistent with their geographic distribution in sect. *Camphora*. For example, *C. camphora*, mainly from Southeast China, is a descendant of the first ancestor; *C. bodinieri* is a descendant of the second ancestor and mainly grows in the upper and middle reaches of the Yangtze River along with its hybrid, *C. septentrionale*; and *C. tenuipilum*, *C. chartophyllum*, *C. mollifolium*, and *Cinnamomum glanduliferum* share the third ancestor and are mainly produced in the Yunnan-Guizhou Plateau. Exceptionally, *C. parthenoxylon* and *C. micranthum* both were inferred as descendants of the third ancestor but are widely distributed in South China. Therefore, we deduced that geographic distribution is one of the important factors affecting speciation of sect. *Camphora* plants and should be considered as a reference to determine the genetic relationship among species. PCA showed that *C. camphora* and *C. parthenoxylon* might be the two most primitive subpopulations within sect. *Camphora* according to the PC2 values (Fig. 2c). The continuous PCA plots also suggested that frequent gene flows possibly occurred between species of sect. *Camphora*. In sect. *Cinnamomum*, the four subgroups were discrete, and an obvious vacancy likely existed between subgroups 2 and 3 because some of the sect. *Cinnamomum* species were not included in this study due to a lack of omics data, or gene flows among species were interrupted for unknown reasons.

Another goal of this study was to identify candidate genes involved in the biosynthesis of the six main terpenoids and the regulatory mechanisms related to the formation of the six chemotypes. TPSs are primarily responsible for terpenoids production, therefore, a comprehensive, genome-wide analysis of the *TPS* gene family was carried out on the basis of the *C. camphora* genome. A total of 78 *TPS* genes were identified and categorized into to six subfamilies. Similar to other Lauraceae species [2, 9], the TPS-b subfamily was also significantly expanded in camphor tree by tandem duplication and segmental duplication events (Table S23, see online supplementary material). Two *TPS* gene clusters occurred on Chr09 and Chr11 in camphor tree, respectively, as well as in *C. kanehirae* [2]. In addition to the six main terpenoids from camphor tree, additional main terpenoids have also been found in other *Cinnamomum* species [17]. The comprehensive survey of the *TPS* gene family in camphor tree would serve as a functional genomic resource for pinpointing the diverse terpenoids biosynthesis in *Cinnamomum.*

*TPS*s are mainly regulated at the transcriptional level in *C. camphora*. In this study, four key *TPS* genes were identified to encode 1,8-cineole, linalool, (iso) nerolidol, and (+)-borneol synthase, respectively. Further, according to an *in vitro* assay, CcTPS16 dominantly produced 1,8-cineole, and its product profile was similar to that of LnTPS1 from *Laurus nobilis* [27]; CcTPS54 was a bi-functional enzyme responsible for both linalool and (iso) nerolidol formation, being parallel to CoLIS from *C. osmophloeum* [28]. Sequence alignment analysis showed that there was no significant difference in the coding sequences of *CcTPS16* and *CcTPS54* among the six chemotypes, which suggests that the major terpenoid contents in LEOs are closely related to the expression of functional *TPS* genes. The differential expression of *TPS* genes among chemotypes might be caused by *cis*-elements in the promoter regions, and *TPS*s were regulated by transcription factors such as ERF [24] and NAC [25]. Subcellular localization of CcTPS54 further showed that cell compartmentalization was also involved in the regulation of the biosynthesis of major terpenoids in *C. camphora*. This regulatory mechanism was also found in other plants [20, 53]. A previous study showed that the TPS-g subfamily contains synthases responding to acyclic terpenoids of floral scent, such as linalool and (iso)nerolidol [19], which is partly consistent with the results of this study. In *C. parthenoxylon*, orthologs of *CcTPS55* from the CcTPS-g subfamily were inferred to be involved in *d*-linalool biosynthesis [54]. Besides, we also speculated that miRNAs might be involved in *CcTPS54* regulation at the post-transcriptional level by small RNA sequencing.

The LEOs of camphor tree are dominated by six main terpenoids, which are indicative of the six chemotypes according to the PCA. In summary, CcTPS16, CcTPS28, CcTPS54, and CcTPS77 were inferred to mediate Cin-, Lin-, Ner-, and Bor-type formation by regulating the content of major terpenoids at the transcriptional level, respectively. Then, CcBHD1 was likely responsive to produce Cam-type from Bor-type by dehydrogenation reaction. Despite low activity *in vitro* [47], *CcBHD1* was up-regulated in the leaves of Cam-type relative to Bor-type and was postulated to produce camphor from (+)-borneol in camphor tree. *CcBHD1* would serve as a genetic target for the production of high-quality (+)-borneol by gene editing. Nevertheless, more biochemical evidence of CcTPS28 and CcTPS77 is required to explore in the future research.

Due to a lack of data, such as seed transcriptome, enzyme catalysis, and heterogeneous expression, it would be necessary to further verify the proteins involved in MCT biosynthesis of camphor tree. The comparative genomics analysis revealed that three genes specifically coding for FatB1, type-B LPAAT, and DGAT2b, respectively, were lost or pseudogenized in LCT-producing species and were inferred to play important roles in MCT accumulation [33, 40, 42]. The results supported the idea that MCT and LCT biosynthesis shared a similar pathway and were regulated by several specific enzymes in a few steps. MCT biosynthesis in Lauraceae is likely regulated at both hydrolysis of acyl-ACP and MCT assembly by specific enzymes but not at carbon chain extension, which is obviously different from that in *Cuphea* [38, 39]. DGAT2 mediates the insertion of MCFAs into the *sn*-3 position of triglyceride, which is also different from that in coconut [43]. These results indicate that MCT biosynthesis in plants is probably mediated by species-specific mechanisms. Besides, with the high-quality *C. camphora* genome assembly constructed in this study, quantitative trait loci that are related to the seed size and oil content should also be investigated by genome-wide association study and genome-wide linkage mapping in the future.

In conclusion, we assemble a chromosome-level genome of *C. camphora*, which is 706.34 Mb in length and harbors 36 411 protein-coding genes. This genome assembly, in combination with the whole-genome resequencing data of 24 *Cinnamomum* species, provides a platform for elucidating the genetic relationship, chemodiversity, and MCT biosynthesis among *Cinnamomum* species*.* This high-quality reference genome would lay a solid foundation for molecular breeding of *C. camphora.*

## Materials and methods

### Sample preparation and sequencing

#### *De novo* genome sequencing

Fresh leaves of camphor tree (Lin-type) were collected from Jiangxi Academy of Forestry, Jiangxi Province, China, and genomic DNA was extracted with a modified cetyltrimethylammonium bromide method. Paired-end (PE) libraries with an insert size of 170–500 bp and mate-pair libraries with a insert size of 2–20 kb were prepared following the Illumina standard protocols and sequenced on an Illumina HiSeq 2000 platform (Illumina, San Diego, California, USA). SMRT libraries with an insert size of 20 kb were constructed according to the PacBio 20-kb protocol (https://www.pacb.com/) and 10 SMRT cells were loaded on Pacbio Sequel II system (Pacific Biosciences of California, Menlo Park, California, USA).

#### Hi-C sequencing

To capture the interacting DNA segments, 3 g of seedlings were crosslinked with 2% formaldehyde solution. Then, chromatin was extracted and digested with the *Dpn*II restriction enzyme. Subsequently, a DNA library was constructed with an insert size of 300–700 bp and sequenced on an Illumina HiSeq 2500 platform (Illumina, San Diego, California, USA) to obtain 150 bp PE reads.

#### Whole-genome resequencing of Asian *Cinnamomum* species

Plant materials used in this study were collected from Xishuangbanna Tropical Botanical Garden, Chinese Academy of Sciences, Yunnan Province, China, and Jiangxi Academy of Forestry. DNA libraries with an insert size of 350 bp were constructed and sequenced on an Illumina NovaSeq 6000 (Illumina, San Diego, California, USA) with a 150 bp PE strategy. Raw reads were filtered based on the following criteria: PE reads with >10% ‘N’ bases; reads with >50% of the bases having a phred quality score <20; and sequencing adapters were removed.

#### Transcriptome sequencing

The Quick RNA Isolation Kit was used to extract total RNA from mature leaves of six chemotypes. Three sets of libraries were constructed and sequenced. The first set of libraries had an average insert size of 200 bp and were sequenced on an Illumina HiSeq 2000 platform to assess the quality and completeness of the genome assembly. The second set of libraries were sequenced on the PacBio Sequel II platform to assist in the identification of protein-coding genes. Finally, to investigate the genes involved in the regulation of terpene biosynthesis, 18 libraries from six chemotypes were sequenced on an Illumina HiSeq 2500 platform to generate DGEs.

#### miRNA sequencing

Five small RNA sequencing libraries were created from mature leaves of five chemotypes according to the manufacturer’s instructions and sequenced on an Illumina HiSeq platform (Illumina, San Diego, California, USA) using the recommended protocol by the manufacturer to produce single-end reads (50 bp).

### Genome size estimate and assembly

To estimate the genome size of *C. camphora*, all the clean PE reads of 170–500 bp were subjected to calculate 19-mer frequency distribution using jellyfish v2.27 [55]. Then, the genome size and heterozygosity rate based on the 19-mer frequency histogram were estimated by GenomeScope v2.0 [56].

Errors in the original PacBio data were corrected using Canu v1.9 [57]. Then, genome assembly and haplotype separation were performed by FALCON [58]. The Arrow consensus caller (https://github.com/PacificBiosciences/GenomicConsensus) was used to generate the polished contigs to improve assembly quality. SSPACE v3.060 was used to scaffold the contigs using mate-pair reads [59]. Pilon v1.2261 was used to eliminate InDel and SNP errors to improve assembly quality using whole-genome sequencing clean PE reads [60]. TrimDup (Rabbit Genome Assembler: https://github.com/gigascience/rabbit-genome-assembler) was used to remove redundant haplotype contigs generated due to the high level of heterozygosity in the *C. camphora* genome.

The quality and completeness of the genome assembly were assessed in several ways. Firstly, five BAC clones of *C. camphora* were sequenced by Sanger sequencing and used as reference sequences. Then the assembled scaffolds were aligned to these reference sequences using BLAST v2.2.25 [61] (−p blastn -e 1e-20) to check the coverage rate. Secondly, BUSCO v3 [62] was used to assess the completeness of the assembled scaffolds by mapping plant conserved orthologous genes (embryophyta_odb10) to these scaffolds. Thirdly, the RNA-seq reads were assembled into unigenes by Trinity v2.4.0 [63] and mapped onto the assembled scaffolds using BLAST v2.2.25 [61] with a 90% identity cutoff to confirm the coverage of the assembly.

One Hi-C library was produced and sequenced to generate clean data, which were truncated firstly at the putative Hi-C junctions and then mapped to the assembly with the MEM algorithm of BWA v0.7.10-r789 [64]. Only uniquely mapped PE reads (mapping quality >20) were retained for further analysis by HiC-Pro v2.8.1 [65]. Finally, valid interaction pairs were extracted and used for correction of scaffolds, and clustered, ordered, and orientated scaffolds onto chromosomes by LACHESIS software [66].

### Characterizations of repetitive sequences

Repetitive sequences were annotated using a combination of homology-based and *de novo* prediction approaches. For homology-based identification, we used RepeatMasker and RepeatProteinMask v4.0.7 [67] to search against the Repbase database v21.12 [68] to identify transposable elements. For *de novo* identification, a *de novo* consensus repeat database was first built using RepeatModeler v1.0.11 (http://www.repeatmasker.org/RepeatModeler) and LTR_FINDER v1.0.6 [69]. Then RepeatMasker was used to identify repeat elements based on the newly built database. Finally, we combined repeat elements obtained from both *de novo* and homolog-based predictions according to the coordination on the chromosomes. In addition, we annotated the tandem repeats using TRF v4.04 [70].

### Gene prediction and annotation

Gene prediction was performed using a combination of methods involving *de novo* prediction, homology-based prediction (using proteins from *Arabidopsis thaliana*, *C. kanehirae*, *Populus trichocarpa*, *Vitis vinifera*, and *Amborella trichopoda*), and unigenes assembled from transcriptome data. MAKER v2.31.8 [71] was used to annotate and integrate gene models, with homologous proteins as an input. The assembled unigenes were aligned to the genome assembly of *C. camphora* using Exonerate v2.2.0 [72] (est2genome), whereas the PacBio Iso-seq reads were polished first using Quiver (https://github.com/PacificBiosciences/GenomicConsensus) and then mapped to the genome by GMAP [73] (parameters: –min-intronlength = 10 –max-intronlength-ends = 50 000 —no-chimeras). For *de novo* prediction, Augustus v3.2.1 [74] and SNAP v2006-07-28 [75] were trained with nearly full-length intact genes of *C. camphora* (intact structure: start codon, stop codon, and perfect intron-exon boundary), which were obtained from the above-mentioned gene models. Then, the two trained programs were used to perform *de novo* prediction on the repeat-masked genome using the MAKER pipeline. To assess the quality of gene annotation, AED scores were generated for each of the predicted genes as part of the MAKER pipeline. We filtered out the low-quality genes that met the following criteria: (1) contain premature stop codon; and (2) coding sequence length < 90 bp. Additionally, we also refined and complemented some functional genes by manual curation based on homology information. Genome annotation completeness was assessed using BUSCO v3 [62] software with the embryophyta_odb10 database.

To obtain functional annotation for the coding genes, the amino acid sequences of the coding genes were aligned against the KEGG (release 87), SWISS-PROT (release-2018_07), TrEMBL (release-2018_07), NR (release 20 170 924), and KOG (release 20 090 331) databases using BLAST v2.2.26 [61] (−p blastp -e 1e-05), and the best match from the alignment was assigned to represent the gene function. GO functional information [76] was retrieved from NR by converting the NR accession IDs to GO terms.

### Genome-evolution analysis

Gene sets of 18 additional angiosperms (Table S18, see online supplementary material) were used for genome-wide evolutionary analysis. All gene sets were processed using the following criteria: (i) elimination of genes with an internal stop codon in the coding sequence; (ii) the longest mRNA was kept if a gene had multiple alternative splicing transcripts; and (iii) if a gene had symbols of mix-bases, the mixed bases were recorded to NNN for the codon, and the corresponding amino acid was coded to X.

OrthoFinder v2.3.11 [77] was used to identify orthogroups by setting the MCL inflation value to 1.5; other parameters were default settings. Genes belonging to orthogroups, genes specific to *C. camphora*, and single-copy orthogroups were all identified. Afterward, phylogenetic trees were constructed based on single-copy orthologous genes and LCN genes in all 19 species with concatenation and coalescent-based approaches, respectively. In each orthogroup, the amino acid sequences were first aligned using MAFFT v7.310 [78]. The alignment was converted into corresponding DNA sequences by PAL2NAL v14 [79]. Next, trimAl v1.4 [80] was used to trim low-quality alignment regions with the option ‘-automated 1’. Then, a gene tree of each orthogroup was constructed by IQ-TREE v1.6.12 [81] (−m MFP -B 1000) coupled with ModelFinder [82] to select the best-fit model. Finally, the coalescent-based species trees based on single-copy orthologous and LCN genes were inferred by ASTRAL v5.7.8 [83], respectively, and the species tree based on the concatenation method was constructed using IQ-TREE and single-copy orthologous genes. We also employed STAG [84] to infer phylogenetic trees based on LCN genes.

To estimate the evolutionary timescale of these species, the Markov chain Monte Carlo algorithm for Bayes estimation was adopted to estimate the divergence time using the program MCMCTree of the PAML package [85], with a burn-in of 500 000 iterations, based on single-copy orthologous genes. Five carefully vetted fossil-based divergence dates from previous studies and TimeTree (http://www.timetree.org) were used for calibration.

We used CAFÉ v3.0 [86] to analyse the expansion and contraction of gene families based on the constructed phylogenetic tree of the 19 species, with the predicted divergence time. Gene families with a tremendous change in size (≥200 genes in one species and ≤2 in all other species) and the most recent common ancestor size equaling to 0 predicted by the parsimony method were discarded.

### Whole-genome synteny and duplication analyses

To identify colinearity in these genomes, all-versus-all BLASTP v2.2.26 [61] (−e 1e-10 -b 5 -v 5) was used to detect paralogs within and among species. Then, syntenic blocks within and among species were identified using MCScanX [87] (−k 50 -s 5 -e 1e-05 -m 25 -a). We extracted all the paralogous and orthologous gene pairs from syntenic blocks to further calculate the 4DTv distance using the HKY substitution model [88]. We also calculated the *Ks* of gene pairs in syntenic blocks using the Nei-Gojobori method implemented in the yn00 program of PAML [85].

### Analysis of the genetic relationship of *Cinnamomum* species via whole-genome resequencing

All clean reads were mapped to the *C. camphora* genome by BWA [65]. The duplicate reads were marked using PICARD v1.94 (http://broadinstitute.github.io/picard) and SAMtools v1.3.1 [89]. Local re-alignments around the InDel regions were performed by InDel-Realigner in GATK v3.8 [90]. SNPs and InDels were called using the HaplotypeCaller module in GATK and were filtered with the following parameters: QD < 2.0, MQ < 40.0, FS > 60.0, QUAL <30.0, MQrankSum < −12.5, ReadPosRankSum < −8.0, -clusterSize 2, and -clusterWindowSize 5. The SNPs identified by GATK were further filtered: SNPs with a minor allele frequency of >5% and missing data <90% were considered high-confidence SNPs. Annotation of SNPs was performed on the basis of the *C. camphora* genome using SnpEff [91], and SNPs were categorized into intergenic regions, upstream or downstream regions, and exonic or intronic regions. SNPs in coding regions were further classified as synonymous or nonsynonymous SNPs. InDels located in exons were grouped according to whether they led to a frameshift.

To analyse the phylogenetic relationship of Asian *Cinnamomum* species, we constructed an unrooted phylogenetic tree using the Mrbayes method [92], and corroborated with a maximum likelihood tree by IQ-TREE2 software [93] (v2.1.3, −m MFP -B 1000) with the best-fit model and 1000 replicates. The chronos program from the R package ape [94] was used for speciation time inference, with the parameter setting model = ‘correlated’ and lambda = 1. Then, ADMIXTURE v1.22 [95] was used to infer the population structure based on high-confidence SNPs, with *K* values (the putative number of populations) ranging from 1 to 10. We assessed the number of sub-populations using five-fold cv. The bar plot of Q matrix for each *K* value stacked assignment was generated using the R package ‘pophelper’ [96]. PCA of the SNPs was performed using the smartpca program [97] with default parameters.

### Identification of miRNAs involved in terpenoid biosynthesis

Firstly, the program ACGT101-miR (LC Sciences, USA) was used to remove adapters, short reads, low-quality sequences, repeats (v18.02; http://www.girinst.org/repbase), mRNAs (RNA-seq data in this study), and common non-coding RNAs (v11; http://rfam.janelia.org) from the raw reads. Then, the remaining clean reads of 18–25 nt were mapped to the *C. camphora* genome to determine genomic locations using Bowtie2 [98] and aligned against the miRBase database (v21; ftp://mirbase.org/pub/mirbase/CURRENT/). The known miRNAs were identified by alignments with length variation at both 3′ and 5′ ends and at most two mismatches were allowed. Furthermore, the flanking 120-nt sequences of unmapped sequences from the exon-antisense, intronic, and intergenic regions were extracted from the genome to predict secondary structures using RNAfold (http://rna.tbi.univie.ac.at/cgi-bin/RNAfold.cgi) according to the criteria to predict potential novel miRNAs. The raw reads of miRNAs were normalized among the five chemotypes by global normalization procedures. In view of no biological replicates in each chemotype, miRNAs with an abundance ≥50 or expressed in at least two samples were selected for the following analysis. TargetFinder (https://github.com/carringtonlab/TargetFinder) and psRNATarget [99] were used to identify miRNA binding sites and their target genes with an expectation value of ≤3.

### Gene differential expression analysis

In-house Perl scripts were used to produce clean data, which were then mapped to the *C. camphora* genome assembly by HISAT2 (https://daehwankimlab.github.io/hisat2/). Only reads with a perfect match or one mismatch were further analysed and annotated based on the *C. camphora* genome. Gene expression levels were estimated by FPKM values. The thresholds for significantly differential expression were set at *P*-value <0.01 and log2 (fold-change) ≥1.0.

### Identification and functional analysis of TPSs

The HMM profiles of TPS (PF01397 and PF03936) were obtained from Pfam. HMMER v3.2.1 [100] was used to search for TPS members in the predicted proteome of *C. camphora* using HMM profiles as queries (e-value <10^−5^). The candidate proteins were further inspected manually to confirm the putative full-length based on homologs of *Arabidopsis* by BLASTP [61] (e-value <10^−5^). To avoid missing potential proteins caused by InDel or SNP errors from sequencing, the genome was employed reversely as queries and aligned against candidate protein sets by the BLASTX program [61] (e-value <10^−5^), and candidates were corrected according to the assembled transcriptomes. TBtools [101] was then used to visualize the chromosomal distribution and exon/intron structures of the *TPS* genes. The maximum likelihood trees were built by CIPRES (https://www.phylo.org) with the JTT model using 1000 bootstrap replicates (Data S1, see online supplementary material).

Expression levels of *TPS* genes were evaluated according to the FPKM values from leaf transcriptomes of the six chemotypes and were validated by qRT-PCR analysis [102], with *CcActin* as the reference gene. The DEGs among the six chemotypes were chosen for clustering analysis using a normalized method. Based on the transcripts obtained from Iso-seq (PacBio), *CcTPS16*, *CcTPS28*, *CcTPS54*, and *CcTPS77* were cloned using specific primers (Table S35, see online supplementary material).

For functional analysis *in vitro*, the truncated *CcTPS16* (Genbank accession number OM721572) and *CcTPS54* (OM721573) were inserted into the pET28a vector, respectively, and then transformed into *Escherichia coli* BL21 (DE3) Rosetta cells. Recombinant proteins were harvested and purified using ProteinIso Ni-NTA Resin (Transgen, China) after incubation in 0.3 mM isopropyl dgalactopyranoside (IPTG) for 12 h at 20°C. Then, 1 μg of recombinant proteins were added with 10 mM substrate (GPP or FPP, Sigma), 10 mM MgCl_2_, 10 mM MnCl_2_, 10% (v/v) glycerol, 5 mM DTT, and 50 mM Bis-Tris (pH 7.0) and incubated at 30°C for 1 h. The products were analysed by GC–MS. The retention time was compared with that of an authentic standard (Sigma-Aldrich) to confirm the main products. Other products were identified using the NIST database.

For protein localization observation, the open reading frames of *CcTPS16* and *CcTPS54* were inserted into the pCAMBIA1300-GFP vector, respectively. Then, the recombinant vectors were transformed into *Agrobacterium tumefaciens* strain GV3101 and transiently expressed in *N. benthamiana* leaves*.* Three days after infiltration, GFP fluorescent signal was observed using an Olympus FV3000 confocal laser scanning microscope (Olympus Corporation, Tokyo, Japan) with excitation and emission wave lengths of 484 nm and 507 nm, respectively.

### Molecular basis for MCTs biosynthesis in *C. camphora*

To identify the candidate genes involved in MCT biosynthesis in *C. camphora*, we downloaded reference sequences that were characterized in lipid metabolism from TAIR [103] and specific orthologs from MCT-producing plants as query sequences. BLAST [61] was used to identify orthologs in the *C. camphora* genome. The phylogenetic trees of KASs, ACPs, FATs, LPAATs, and DGATs from MCT-producing plants were constructed to predict preferential functions with the same protocols for TPSs described above (Data S2–S7, see online supplementary material).

SimpleSynteny [104] was applied to compare small syntenic blocks from the Lauraceae genomes to reveal molecular bases responsible for divergent triglyceride biosynthesis. Firstly, candidate genes, as well as their flanking sequences harboring nine other putative genes, were extracted as query syntenic blocks. Then, the corresponding syntenic blocks of *L. cubeba*, *C. kanehirai*, *P. americana*, and *P. bournei* were identified by MCScanX [87] and extracted. Finally, the conservation and completeness of the ten genes in each syntenic block were evaluated and visualized by SimpleSynteny [104].

### PCA of *C. camphora* chemotypes growing in Jiangxi Province

####  

LEO profiles from 250 camphor trees, which were preliminarily divided into five chemotypes according to the scent, were investigated following Qiu *et al* [54]. The top twenty compounds of each individual were chosen and subjected to PCA using Canoco 5 software [105].

## Acknowledgments

This work was financially supported by the Natural Science Foundation for Young Scientists of Jiangxi Province, China (20202ACBL215003), the National Natural Science Foundation of China (31460209), and the Found for Fostering Talent of Jiangxi Academe of Forestry (2017512702). We would like to thank Biomarker Technologies Co., Ltd for their assistance in Hi-C sequencing and whole genome resequencing of *Cinnamomum*, Dr Yi-Cun Chen from the Chinese Academy of Forestry for valuable manuscript suggestions, Dr Yun-Xiao Zhao from the Chinese Academy of Forestry for functional analysis, Dr Zhi-Yong Xie from Huazhong Agricultural University for protein subcellular location, and the functional analysis, Xishuangbanna Tropical Botanical Garden for plant materials.

## Author contributions

X.-M. J. and C. Z. were the leaders of this study. X.-D.W., C.-Y. X., Y.-J. Z. and Y.-F. W. designed the experiments and wrote the manuscript. X.-D.W., Y.-J.Z. and T.Z. performed the whole genome resequencing, transcriptome, and small RNA analyses. C.-Y.X., Z.-Y.X., T.F., H.X., Y.-L.C., H.-Q.W., and Q.-Q.Z. carried out the genome assembly, annotation, and phylogenomic analyses. H.-K.Y., F.-Y.Q., X.-Y.D., X.-S.H., and J.L. extracted essential oils. C.F. and S.-X.L. collected plant material; X.-D.W., Y.-T.Z. and S.-S.Z. performed functional analysis. Y.-D.W. coordinated the project and polished the manuscript writing.

## Data availability

Data supporting the findings of this work were available within the paper and its supplementary information files. The genomic, small RNA, and transcriptome sequencing data in this manuscript have been deposited into the National Center for Biotechnology Information (NCBI) under accession numbers PRJNA753557 and PRJNA799645. The whole-genome resequencing data of Asian *Cinnamomum* species are available in China National GeneBank DataBase (CNGB) (ID CNP0003375).

## Conflict of interest

The authors declared that they had no conflict of interest.

## Supplementary data

Supplementary data is available at *Horticulture Research* online.

## Supplementary Material

Web_Material_uhac216
