# Supplementary figures and images for: Chromosome-level genome assembly and resequencing of camphor tree (Cinnamomum camphora) provides insight into phylogeny and diversification of terpenoid and triglyceride biosynthesis of Cinnamomum
Source: Hortic Res. 2022 Sep 21;9:uhac216. doi: 10.1093/hr/uhac216 (PMC9720445; doi:10.1093/hr/uhac216)

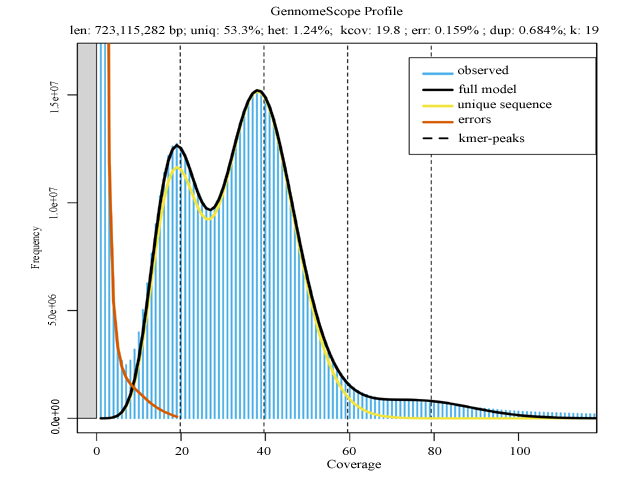

Supplement: Web_Material_uhac216 [file web_material_uhac216.zip › Supplementary Figure 1.tif]

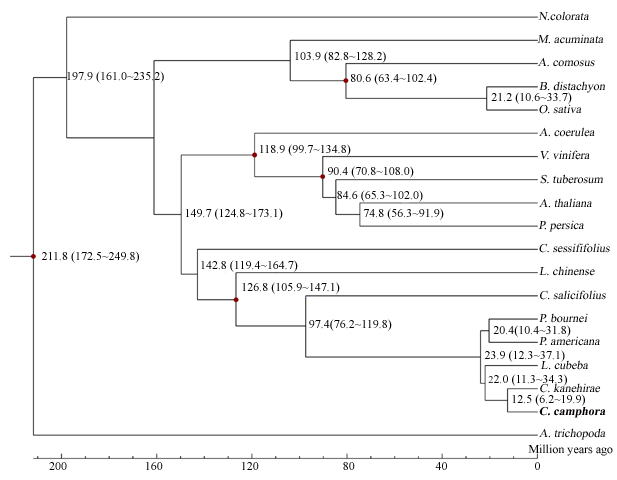

Supplement: Web_Material_uhac216 [file web_material_uhac216.zip › Supplementary Figure 10.tif]

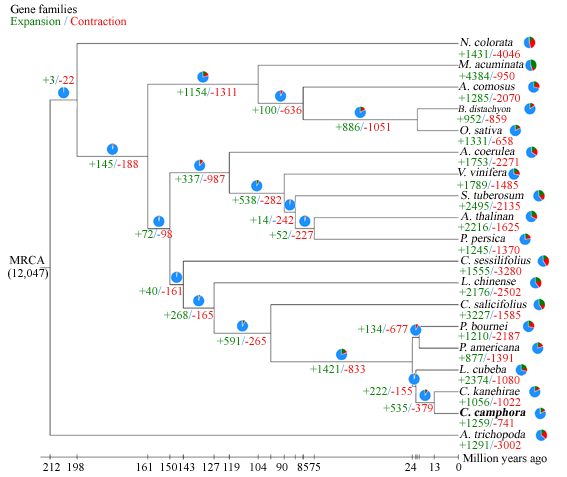

Supplement: Web_Material_uhac216 [file web_material_uhac216.zip › Supplementary Figure 11.tif]

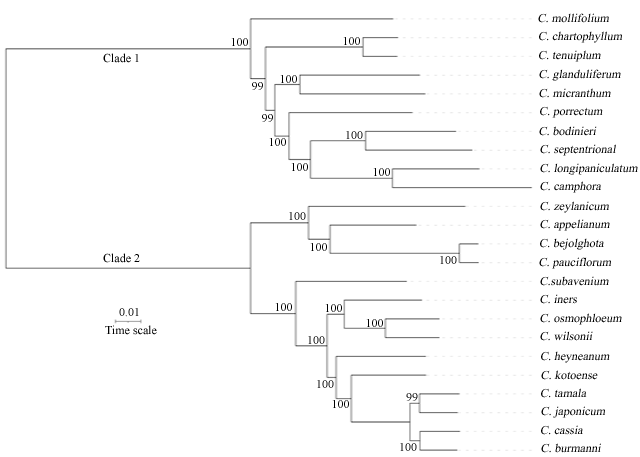

Supplement: Web_Material_uhac216 [file web_material_uhac216.zip › Supplementary Figure 12.tif]

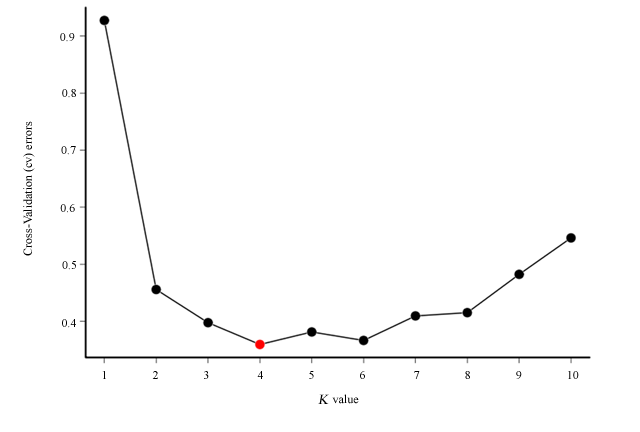

Supplement: Web_Material_uhac216 [file web_material_uhac216.zip › Supplementary Figure 13.tif]

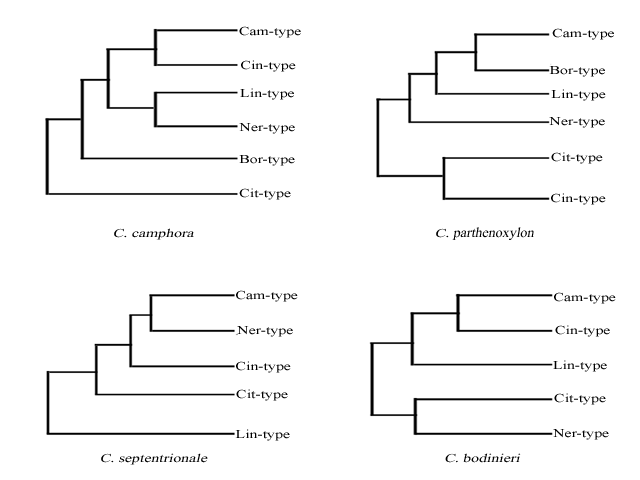

Supplement: Web_Material_uhac216 [file web_material_uhac216.zip › Supplementary Figure 14.tif]

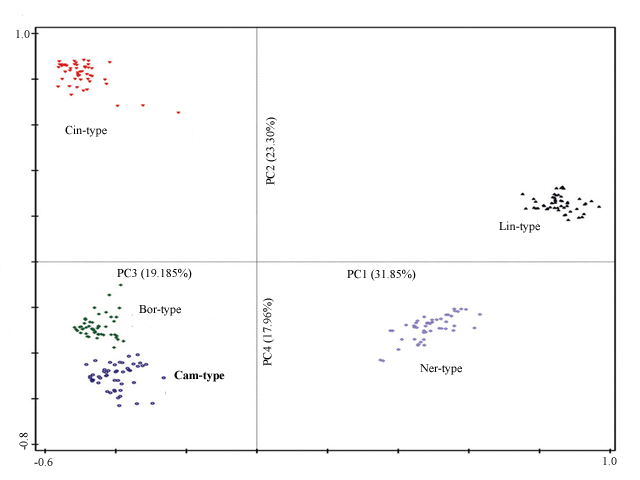

Supplement: Web_Material_uhac216 [file web_material_uhac216.zip › Supplementary Figure 15.tif]

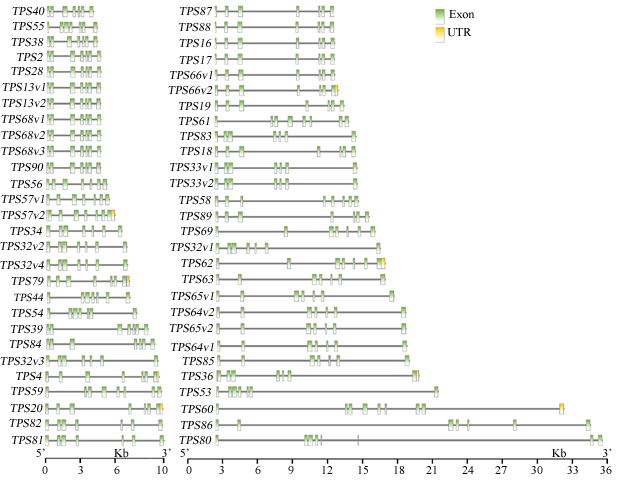

Supplement: Web_Material_uhac216 [file web_material_uhac216.zip › Supplementary Figure 16.tif]

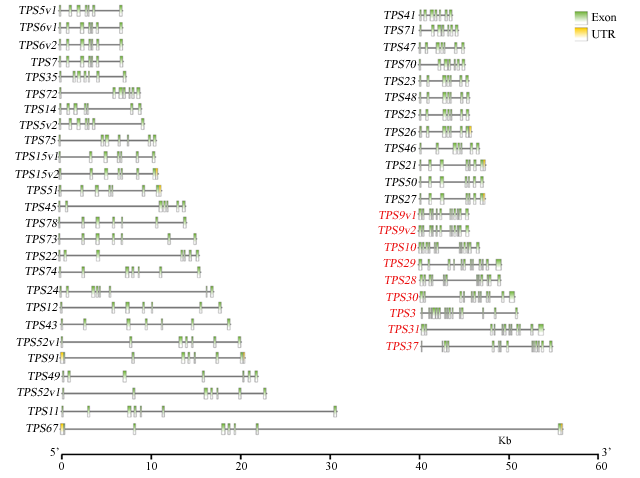

Supplement: Web_Material_uhac216 [file web_material_uhac216.zip › Supplementary Figure 17.tif]

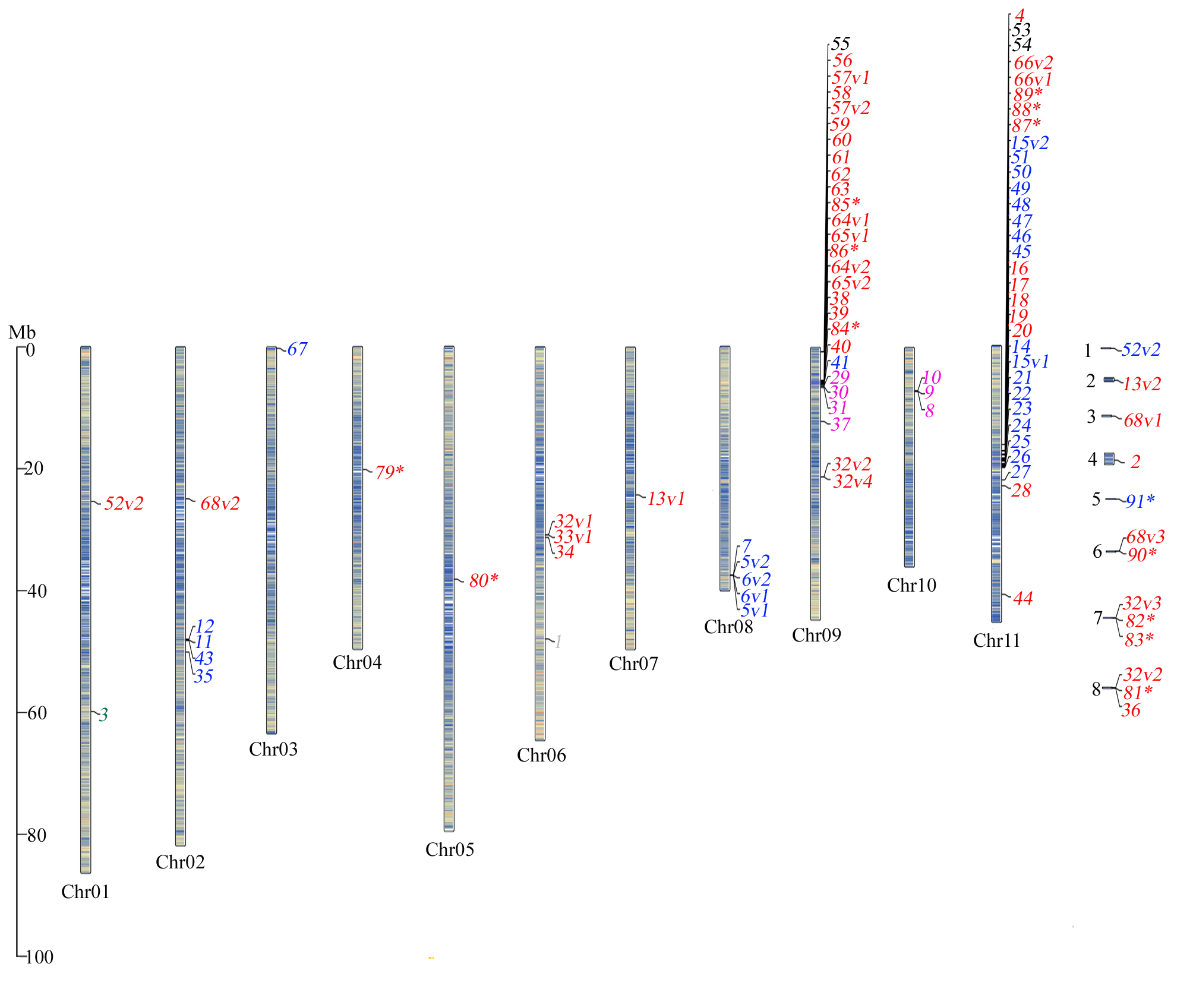

Supplement: Web_Material_uhac216 [file web_material_uhac216.zip › Supplementary Figure 18.tif]

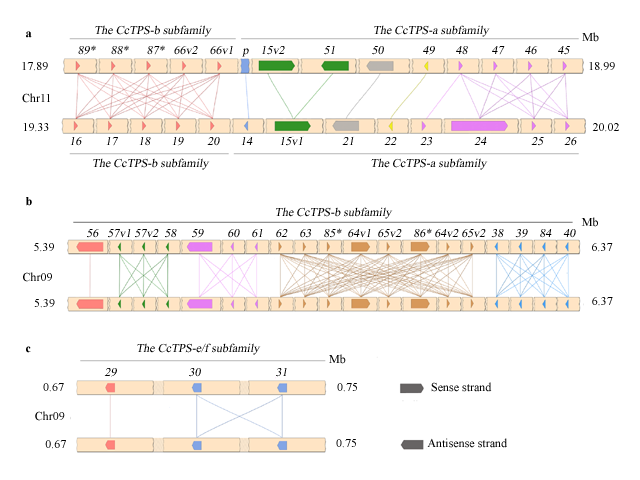

Supplement: Web_Material_uhac216 [file web_material_uhac216.zip › Supplementary Figure 19.tif]

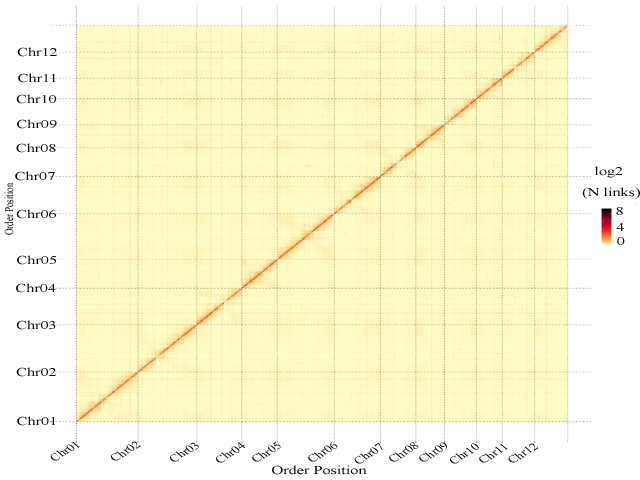

Supplement: Web_Material_uhac216 [file web_material_uhac216.zip › Supplementary Figure 2.tif]

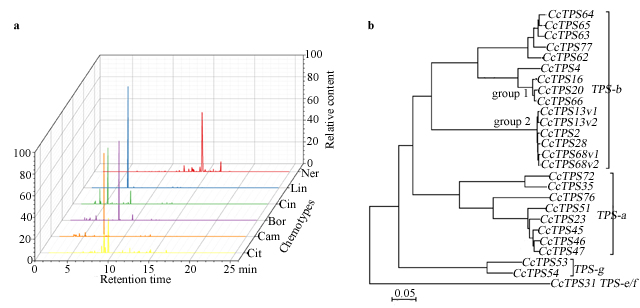

Supplement: Web_Material_uhac216 [file web_material_uhac216.zip › Supplementary Figure 20.tif]

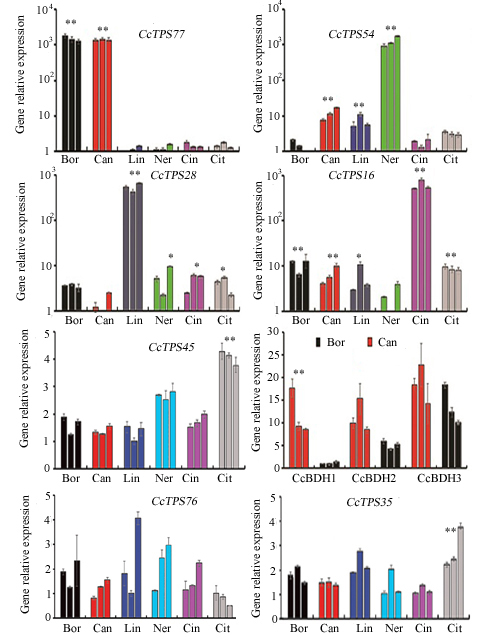

Supplement: Web_Material_uhac216 [file web_material_uhac216.zip › Supplementary Figure 21.tif]

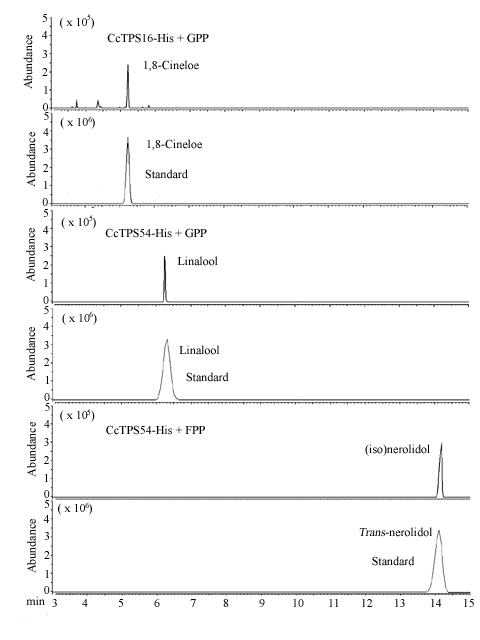

Supplement: Web_Material_uhac216 [file web_material_uhac216.zip › Supplementary Figure 22.tif]

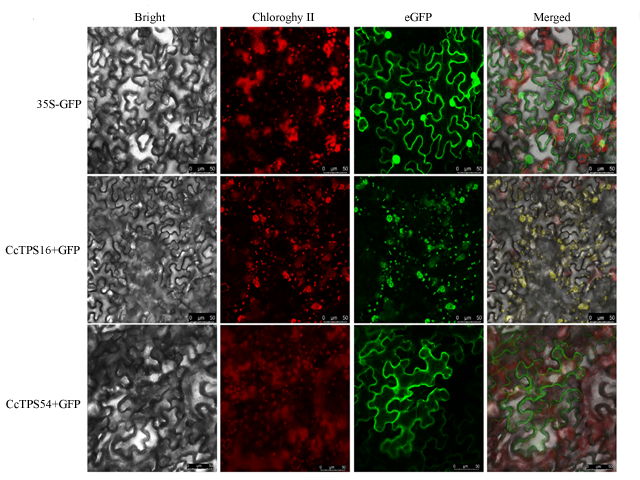

Supplement: Web_Material_uhac216 [file web_material_uhac216.zip › Supplementary Figure 23.tif]

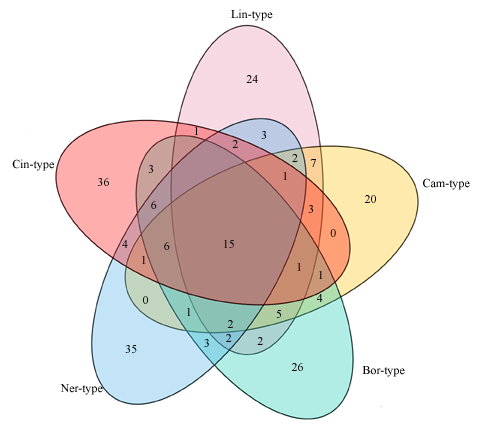

Supplement: Web_Material_uhac216 [file web_material_uhac216.zip › Supplementary Figure 24.tif]

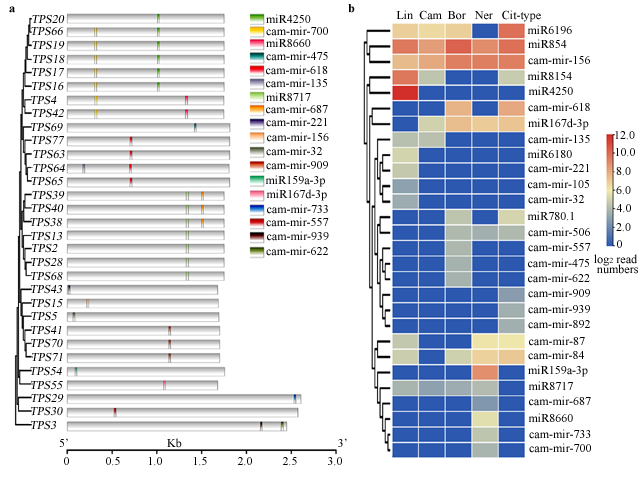

Supplement: Web_Material_uhac216 [file web_material_uhac216.zip › Supplementary Figure 25.tif]

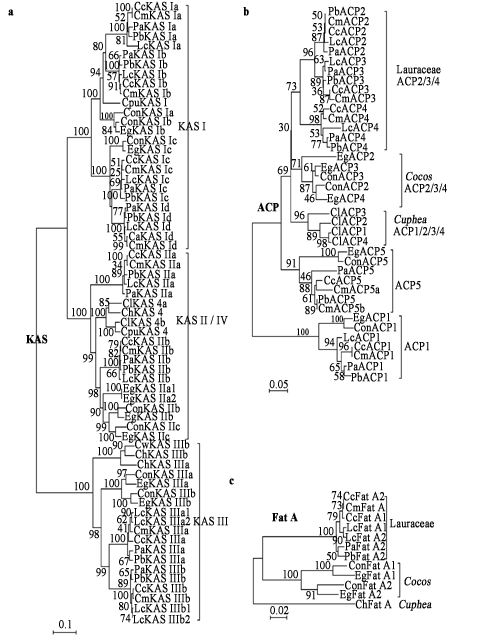

Supplement: Web_Material_uhac216 [file web_material_uhac216.zip › Supplementary Figure 26.tif]

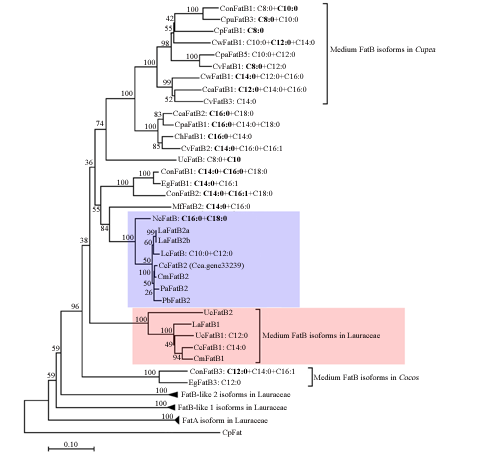

Supplement: Web_Material_uhac216 [file web_material_uhac216.zip › Supplementary Figure 27.tif]

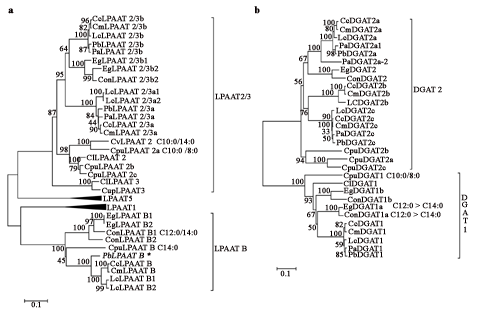

Supplement: Web_Material_uhac216 [file web_material_uhac216.zip › Supplementary Figure 28..tif]

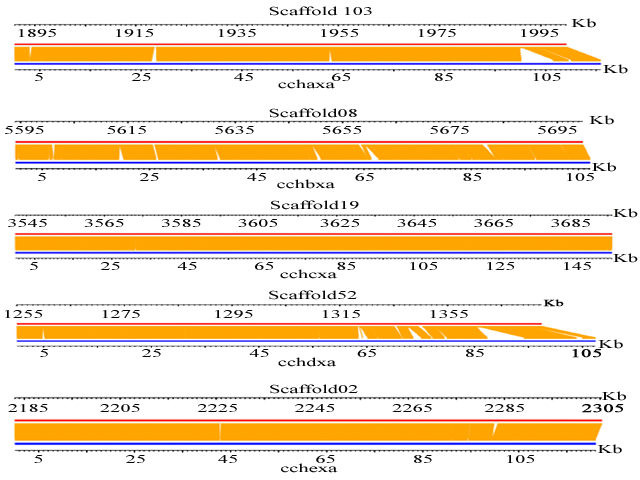

Supplement: Web_Material_uhac216 [file web_material_uhac216.zip › Supplementary Figure 3.tif]

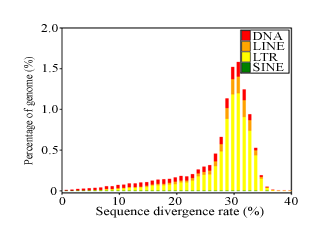

Supplement: Web_Material_uhac216 [file web_material_uhac216.zip › Supplementary Figure 4.tif]

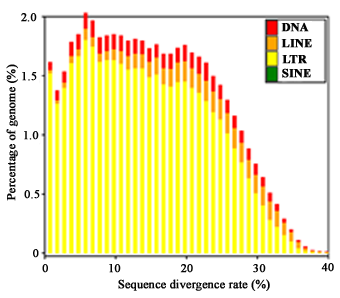

Supplement: Web_Material_uhac216 [file web_material_uhac216.zip › Supplementary Figure 5.tif]

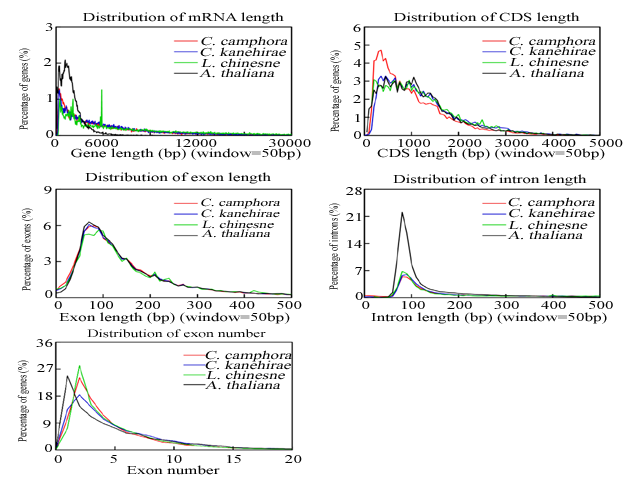

Supplement: Web_Material_uhac216 [file web_material_uhac216.zip › Supplementary Figure 6.tif]

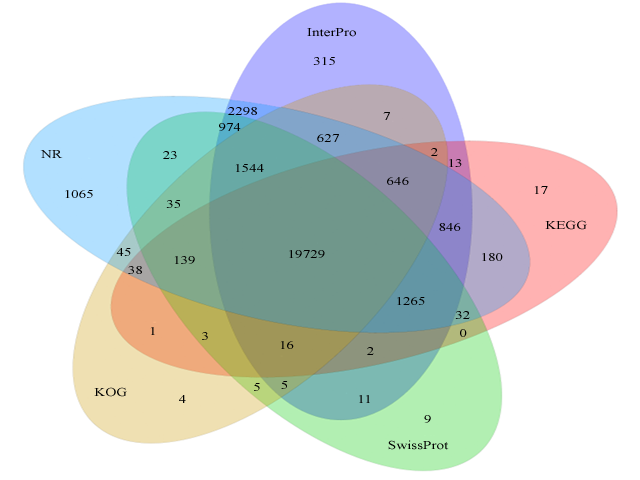

Supplement: Web_Material_uhac216 [file web_material_uhac216.zip › Supplementary Figure 7.tif]

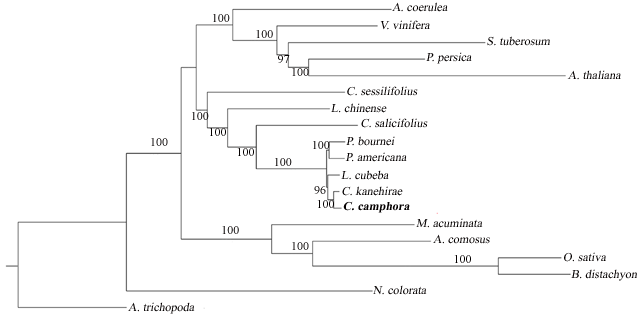

Supplement: Web_Material_uhac216 [file web_material_uhac216.zip › Supplementary Figure 8.tif]

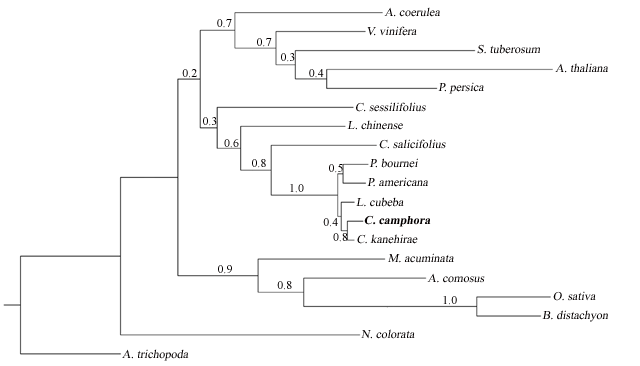

Supplement: Web_Material_uhac216 [file web_material_uhac216.zip › Supplementary Figure 9.tif]
