# Supplementary material for: Chromosome-level genome assembly and resequencing of camphor tree (Cinnamomum camphora) provides insight into phylogeny and diversification of terpenoid and triglyceride biosynthesis of Cinnamomum
Source: Hortic Res. 2022 Sep 21;9:uhac216. doi: 10.1093/hr/uhac216 (PMC9720445; doi:10.1093/hr/uhac216)
Supplement: Web_Material_uhac216 [file web_material_uhac216.zip › Supplementary Figures 1-28.doc]

**Supplementary Figures**

**Chromosome-level genome assembly and resequencing of camphor tree (*Cinnamomum camphora*) provides insight into phylogeny and diversification of terpenoid and triglyceride biosynthesis of *Cinnamomum***

Xin-Dong Wang^1,3^†, Chun-Yan Xu^2^†, Yong-Jie Zheng^1,4^†, Yan-Fang Wu^1^†, Yue-Ting Zhang^1,3^, Ting Zhang^1^, Zhen-Yu Xiong^3^, Hai-Kuang Yang^1^, Jiang Li^1^, Chao FU^1^, Feng-Ying Qiu^1^, Xiao-Ying Dai^1^, Xin-Liang Liu^1^, Xiao-San He^1^, Song-Song, Zhou^1^, Fu-Ming Xiao^3^, Sheng-Xing Li^1^, Tao Fu^2^, Han Xie^2^, Yan-Ling Chen^2^, Qian-Qian Zhang^2^, Hong-Qi Wang^2^, Yang-Dong Wang^4*^, Cheng Zhou^1*^, Xiang-Mei Jiang^1*^

**Institutions**: 1. Camphor Engineering and Technology Research Center of National Forestry and Grassland Administration, Jiangxi Academy of Forestry, Nanchang 330032, China; 2. BGI Genomics, BGI-Shenzhen, Shenzhen 518083, China; 3. Jiangxi Provincial Key Lab for Plant Biotechnology, Jiangxi Academy of Forestry, Nanchang 330032, Jiangxi, China; 4. Research Institute of Subtropical Forestry, Chinese Academy of Forestry, Hangzhou 311400, China.

**Supplementary Fig**. **1**. **19-mers depth distribution of the *C. camphora* genome***.* The x-axis indicated depth of 19-mers and the y-axis indicated the frequency of 19-mers.

**Supplementary Fig**. **2**. **Interchromosomal Hi-C contact map of the *C. camphora* genome***.* The intensity of each pixel represented the number of Hi-C links of 300 kb resolution in the chromosomes. Darker red pixels denoted higher contact probabilities. Most interactions were observed within the chromosomes.

**Supplementary Fig**. **3**. **Alignments for the 5 BAC Clones to assembled scaffolds of the *C. camphora* genome**. The red bars were the scaffold sequences and the blue ones were the BAC clone sequences, on which some gaps were represented by white bars. The yellow polygons represented that in these regions the BAC clone sequences could align well with the scaffold sequences.

**Supplementary Fig**. **4**. **The sequence divergence rate of four different TEs using RepeatMasker annotation**.

## Supplementary Fig. 5. The sequence divergence rate of four different TEs using *de novo* annotation.

## Supplementary Fig. 6. Gene structure prediction results statistics. *C. camphora* compared with genetic elements of related species (*Cinnamomum kanehirae*, *Liriodendron chinense*, and *Arabidopsis thaliana*). Window referred to the length represented by each point on the horizonal coordinate.

**Supplementary Fig**. **7**. **The distribution of gene function annotation in five functional databases**.

**Supplementary Fig**. **8**. **The concatenated super-matrix phylogenetic tree using ASTRAL**. The tree was constructed by IQ-TREE based on single-copy orthologous genes. The values on branches were bootstrap support values.

**Supplementary Fig**. **9**. **The coalescent-based species tree inferred by STAG based on LCN families**. The values on nodes were the support values that calculated by STAG.

**Supplementary Fig**. **10**. **Divergence times between *C. camphora* and other plant species**. The numbers on the nodes were the divergence time from present (million years ago, MYA). The divergence times marked as red points were the calibration times derived from previously published papers.

**Supplementary Fig**. **11**. **The expansion and contraction of gene families**. Gene family expansions were indicated in green, and gene family contractions were indicated in red. The corresponding proportions among the total changes were shown as pie charts using the same colors. Blue portions of the pie charts represented conserved gene families.

**Supplementary Fig**. **12**. **An unrooted phylogenetic tree of the 24 Asian *Cinnamomum* species using the software IQ-TREE2**.

**Supplementary Fig**. **13**. **The Cross-Validation (cv) errors among *K* values**.

**Supplementary Fig**. **14**. **Typologies of chemotypes in 4 species from *Cinnamomum***. Chemotypes were classified according to the main compound of leaf essential oils (saw below). Cam: camphor; Bor: (+)-borneol; Lin: linalool; Cin: 1,8-cineole; Cit: citral; Ner: (iso)nerolidol.

**Supplementary Fig**. **15**. **Five chemotypes were found in a population** **of** **camphor tree by PCA analysis**. This population with 250 individuals were sampled in Jiangxi Province of China, and the top twenty compounds from each individual (Supplementary Table 23) were subject to PCA analysis. Cam: camphor; Bor: (+)-borneol; Lin: linalool; Cin: 1,8-cineole; Ner: (iso)nerolidol.

**Supplementary Fig**. **16**. **Structure of genes coding for monoterpene synthase in the *C. camphora* genome***.* The genes coding for monoterpene synthases had seven exons.

**Supplementary Fig**. **17**. **Structure of genes coding for sesquiterpene synthase and diterpene synthase in the *C. camphora* genome**. The genes coding for sesquiterpene synthases had seven exons and in black. The genes coding for diterpene synthase were in red.

**Supplementary Fig**. **18**. ***TPS* genes** **located in pseudochromosomes of the *C. camphora****.* The members belonged to the *TPS*-*a* subfamliy (green), *TPS-b* subfamliy (red), *TPS-c* subfamliy (blue), *TPS-g* subfamliy (black) and TPS-e/f subfamily (pink), respectively. Pseudogenes were labeled with *. Thirteen *TPS* genes were unanchored.

**Supplementary Fig**. **19**. ***TPS* genes occurred in tandem arrays on Chr09 and Chr11 of camphor tree**. Clusters, due to tandem duplications and segmental duplication, encoded monoterpene synthases (the *CcTPS-b* family), sesquiterpene synthase (the *CcTPS-a* family) and diterpene synthase (the *CcTPS-e/f* family), respectively. Genes with high sequence identity (> 90%) were connected by lines. In the 17.81-22.05 Mb interval of Chr11, members from the *CcTPS-a* family and the *CcTPS-b* family showed mosaic distribution patterns.

**Supplementary Fig**. **20**. **Cluster analysis of** [**differentially**](javascript:;) [**expressed**](javascript:;) ***TPS* genes among chemotypes on the basis of sequence similarity**. **a**, The GC-MS total ion chromatogram of LEOs from six chemotypes which were subjected to transcriptomes analysis. **b**, Phylogenetic tree of the differentially expressed *TPS* genes among six chemotypes.

**Supplementary Fig**. **21**. **Expression analysis of *TPS* genes using qRT-PCR**. The relative expression levels were estimated from the threshold of the PCR cycle with the delta CT method. The error bars indicate the standard errors of three biological replicates. The columns with "**" had a statistical difference (p < 0.01) when compared to control calculated by Duncan's multiple range test.

**Supplementary Fig**. **22**. **The authentic standard used for functional analysis of CcTPS in GC-MS**.

**Supplementary Fig**. **2****3**. **Subcellular localization of CcTPS16 and CcTPS54 in *N. benthamiana* leaves**. Transient expression of CcTPS16- and CcTPS54-GFP fusions were compared to p35S-GFP as a control in *N*. benthamiana leaves. The adaxial leaf surface was observed with laser confocal microscopy (Bars = 50 µm). eGFP, green fluorescent protein fluorescence image; chlorophyll, chlorophyll autofluorescence image; bright, transmission image; merged, all channels (eGFP, chlorophyll, and bright) combination.

**Supplementary Fig**. **24**. **Distribution of** **miRNAs involved in terpenoid biosynthesis in leaf among five chemotypes**. Lin-type: linalool-type; Cin-type: 1,8-cineole; Cam-type: camphor-type; Bor-type: borneol-type; Ner-type: (iso)nerolidol-type.

**Supplementary Fig**. **25**. **Identification of miRNAs involved in terpenoid biosynthesis in camphor tree leaf by small RNA sequencing**. **a**, Prediction of miRNA in leaf of *C.camphora* with *TPS* as their targeted genes. **b**, Cluster analysis of miRNA involved in terpenoid biosynthesis in leaf among five chemotypes according to expression levels.

**Supplementary Fig**. **26**. **Phylogenetic analysis of ACPs (a), KASs (b) and FatAs (c) in MCT-producing plants**. The deduced amino acid sequences for the phylogenetic tree could be found in Supplementary Data 2-4. ***Cuphea pulcherrima***: CpuKAS I (Genbank accession number: [DQ141237](https://www.ebi.ac.uk/ena/browser/view/DQ141237)), CpuKAS4 ([AF060518](https://www.ebi.ac.uk/ena/browser/view/AF060518)); ***Cocos nucifera***: CocnKAS Ic (JX275887), CocnKAS Ia (KAG1334367.1), CocnKAS Ib (KAG1358842.1), CocnKAS IIb (KAG1342586.1), CocnKAS IIc (KAG1366411.1), ConACP1 (KAG1334379.1), ConACP2 (KAG1371219.1), ConACP3 (EHA8592603.1), ConACP5 (EHA8589480.1), ConFatA1 (MH158655), ConFatA2 (MH158656); ***Elaeis guineensis***: EgKAS Ic (XP_010915981.1), EgKAS Ib (XP_010932377.1), EgKAS IIa1 ([AF220453](https://www.ebi.ac.uk/ena/browser/view/AF220453)), EgKAS IIb (XP_010937286.1), EgKAS IIc (XP_010939869.1), EgKAS IIa2 (AAF26738.2), EgACP1 ([XP_010917412.1](https://www.ncbi.nlm.nih.gov/protein/XP_010917412.1)), EgACP2 (XP_010904753.1), EgACP3 (XP_010921727.1), EgACP4 (XP_010938698.1), EgACP5 (XP_010929652.1), EgFatA1 (XP_010926746); ***C. lanceolata***: ClKAS4a (AJ344250), ClKAS4b ([KC142140](https://www.ebi.ac.uk/ena/browser/view/KC142140)), ClACP1 (X77620); ClACP2 (X77621); ClACP3 (X77622); ClACP4 (X95253); ***C. hookeriana***: ChKAS4 ([AF060519](https://www.ebi.ac.uk/ena/browser/view/AF060519)), ChFATA2 ([AF062399](https://www.ebi.ac.uk/ena/browser/view/AF062399)).

**Supplementary Fig**. **27**. **Phylogenetic analysis of FatB isoforms in MCT-producing plants**. The deduced amino acid sequences for the phylogenetic tree could be found in Supplementary Data 5. ***C. camphora***: CcFat B1 (Genbank accession number: AAC49151); ***C. nucifera***: ConFat B1 (AEM72519), ConFat B2 (AEM72520), ConFat B3 (AEM72521); ***C. calophylla***: CcaFat B1 (ABB71579), CcaFat B2 (ABB71581); ***C. hookeriana***: ChFat B1 (AAC48990), ChFat B2 (AAC49269); ***C. palustris***: CpFat B1 (AAC49179); ***C. paucipetala***: CpaFat B1 (ADR51161), CpaFat B5 (AGL08248); ***C. pulcherrima***: CpuFat B3 (AGG79285); ***C. wrightii***: CwFat B1 (AAC49783), CwFat B2 (AAC49784); ***C. viscosissima***: CvFat B1 (AEM72522), CvFat B2 (AEM72523), CvFat B3 (AEM72524); ***E. guineensis***: EgFat B1 (AAD42220), EgFat B3 (XP_010925110); ***Lindera communis***: LcoFat B (AHF72806); ***Neocinnamomum caudatum***: NcFat B (MG763172); ***Umbellularia californica***: UcFat B1 (AAA34215), UcFat B2 (AAC49001); ***Ulmus americana***: UaFat B1 (AAB71731); ***Myristica fragrans***: MfFat B2 (AAB71729); ***Clostridium perfringens***: CpeFat B1 (ABG82470).

**Supplementary Fig**. **28**. **Phylogenetic analysis of the LPAAT (a) and DGAT (b) family in MCT-producing plants**. The deduced amino acid sequences for the phylogenetic tree could be found in Supplementary Data 6 and 7. ***E. guineensis***: EgLPAAT 1 (Genbank accession number: XP_010908895), EgLPAAT 2/3b1 (XP_010908896), EgLPAAT 2/3b2 (XP_010936132), EgLPAAT 4/5 (XP_010906759), EgLPAAT B1 (XP_010909860), EgLPAAT B2 (XP_010919015), EgDGAT 1a (XP_010924968), EgDGAT 1b (XP_010925472), EgDGAT2 (XP_010932136); ***C. nucifera***: ConLPAAT 1 (KAG1354715), ConLPAAT 2/3b2 (KAG1367020), ConLPAAT B1 (AAC49119), ConLPAAT B2 (KAG1365335), ConDGAT 1a (AZZ09170), ConDGAT 1b (KAG1335378.1); ***C. viscosissima***: CvLPAAT2 (ALM22867); ***C. lanceolata***: ClLPAAT 2 (AHU86564), ClLPAAT 3 (AHU86563), ClDGAT 1 (AHU86562); ***C. pulcherrima***: CpuLPAAT 1 (ALM22868), CpuLPAAT 2a (ALM22869), CpuLPAAT 2b (ALM22870), CpuLPAAT 2c (ALM22871), CpuLPAAT 3 (ALM22872), CpuLPAAT B (ALM22873), CpuDGAT 1 (ANN46862), CpuDGAT 2A (ANN46863), CpuDGAT 2B (ANN46864), CpuDGAT 2C (ANN46865).
